# Supplementary material for: Social mixing patterns in the UK following the relaxation of COVID-19 pandemic restrictions, July–August 2020: a cross-sectional online survey
Source: BMJ Open. 2022 Dec 13;12(12):e059231. doi: 10.1136/bmjopen-2021-059231 (PMC9748508; doi:10.1136/bmjopen-2021-059231)
Supplement: Author's manuscript [file bmjopen-2021-059231.draft_revisions.pdf]

BMJ Open is committed to open peer review. As part of this commitment we make the peer review history of every article we publish publicly available.

When an article is published we post the peer reviewers' comments and the authors' responses online. We also post the versions of the paper that were used during peer review. These are the versions that the peer review comments apply to.

The versions of the paper that follow are the versions that were submitted during the peer review process. They are not the versions of record or the final published versions. They should not be cited or distributed as the published version of this manuscript.

BMJ Open is an open access journal and the full, final, typeset and author-corrected version of record of the manuscript is available on our site with no access controls, subscription charges or pay-per-view fees (http://bmjopen.bmj.com).

If you have any questions on BMJ Open's open peer review process please email
info.bmjopen@bmj.com

# Social mixing patterns in the UK following the relaxation of COVID-19 pandemic restrictions: a cross-sectional online survey.


| | |
|---|---|
| Journal: | *BMJ Open* |
| Manuscript ID | bmjopen-2021-059231 |
| Article Type: | Original research |
| Date Submitted by the Author: | 14-Jan-2022 |



| | |
|---|---|
| Complete List of Authors: | Bridgen, Jessica; Lancaster University, Lancaster Medical School<br>Jewell, Christopher ; Lancaster University Faculty of Health and Medicine, Lancaster Medical School<br>Read, Jonathan; Lancaster University Faculty of Health and Medicine, Lancaster Medical School |



| | |
|---|---|
| Keywords: | COVID-19, EPIDEMIOLOGY, Epidemiology < INFECTIOUS DISEASES, Public health < INFECTIOUS DISEASES |


SCHOLARONE™
Manuscripts

*I, the Submitting Author has the right to grant and does grant on behalf of all authors of the Work (as defined in the below author licence), an exclusive licence and/or a non-exclusive licence for contributions from authors who are: i) UK Crown employees; ii) where BMJ has agreed a CC-BY licence shall apply, and/or iii) in accordance with the terms applicable for US Federal Government officers or employees acting as part of their official duties; on a worldwide, perpetual, irrevocable, royalty-free basis to BMJ Publishing Group Ltd ("BMJ") its licensees and where the relevant Journal is co-owned by BMJ to the co-owners of the Journal, to publish the Work in this journal and any other BMJ products and to exploit all rights, as set out in our licence.*

*The Submitting Author accepts and understands that any supply made under these terms is made by BMJ to the Submitting Author unless you are acting as an employee on behalf of your employer or a postgraduate student of an affiliated institution which is paying any applicable article publishing charge ("APC") for Open Access articles. Where the Submitting Author wishes to make the Work available on an Open Access basis (and intends to pay the relevant APC), the terms of reuse of such Open Access shall be governed by a Creative Commons licence – details of these licences and which Creative Commons licence will apply to this Work are set out in our licence referred to above.*

*Other than as permitted in any relevant BMJ Author's Self Archiving Policies, I confirm this Work has not been accepted for publication elsewhere, is not being considered for publication elsewhere and does not duplicate material already published. I confirm all authors consent to publication of this Work and authorise the granting of this licence.*

For peer review only

# Social mixing patterns in the UK following the relaxation of COVID-19 pandemic restrictions: a cross-sectional online survey

Jessica RE Bridgen; Lancaster Medical School, Lancaster University, Lancaster, UK, LA1 4YW
Chris P Jewell; Lancaster Medical School, Lancaster University, Lancaster, UK, LA1 4YW
Jonathan M Read; Lancaster Medical School, Lancaster University, UK, LA1 4YW
jonathan.read@lancaster.ac.uk (corresponding author)

Word Count: 3228

For peer review only

**Abstract**

**Objectives:** To quantify and characterize non-household contact and to identify the effect of shielding and isolating on contact patterns.

**Design:** Cross-sectional study.

**Setting and participants:** Anyone living in the UK was eligible to take part in the study. We recorded 5,143 responses to the online questionnaire between 28 July and 14 August 2020.

**Outcome measures:** Our primary outcome was the daily non-household contact rate of participants. Secondary outcomes were propensity to leave home over a 7 day period, whether contacts had occurred indoors or outdoors locations visited, furthest distance travelled from home, ability to socially distance, and membership of support bubble.

**Results:** The mean rate of non-household contacts per person was 2.9 $d^{-1}$. Participants attending a workplace (adjusted incidence rate ratio (aIRR) 3.33, 95%CI 3.02 to 3.66), self-employed (aIRR 1.63, 95%CI 1.43 to 1.87) or working in healthcare (aIRR 5.10, 95%CI 4.29 to 6.10) reported significantly higher non-household contact rates than those working from home. Participants self-isolating as a precaution or following Test and Trace instructions had a lower non-household contact rate than those not self-isolating (aIRR 0.58, 95%CI 0.43 to 0.79). We found limited evidence that those shielding had reduced non-household contacts compared to non-shielders.

**Conclusion:** The daily rate of non-household interactions remains lower than pre-pandemic levels, suggesting continued adherence to social distancing guidelines. Individuals attending a workplace in-person or employed as healthcare professionals were less likely to maintain social distance and had a higher non-household contact rate, possibly increasing their infection risk. Shielding and self-isolating individuals required greater support to enable them to follow the government guidelines and reduce non-household contact and therefore their risk of infection.

For peer review only

**Strengths and Limitations**

- This study provides quantitative insight into the social mixing patterns in the UK at the beginning of the second wave of SARS-CoV-2 infection.
- This study provides an estimate of the level of social mixing to support epidemic growth of the contemporary variant.
- This study characterises the ability of those shielding and self-isolating to minimise their contact with others outside of their household and to adhere to government guidelines.
- Certain demographic groups are underrepresented in this study, including the very elderly.
- Social contacts were self-reported by participants and were therefore subject to recall bias.

For peer review only

## INTRODUCTION

On 31 January 2020, the first two cases of COVID-19 were recorded in the United Kingdom (UK), followed by a rapid rise in identified cases and hospitalised patients. On 23 March 2020, a range of social distancing measures were implemented across the UK (lockdown), aiming to reduce interpersonal contact between households and reduce transmission of SARS-CoV-2. Schools were closed to pupils, with the exception of children of key workers. People were only allowed to leave their homes to shop for basic necessities, to exercise once a day, for medical reasons, and to travel to work if working from home was not possible.[1] By July 2020, many businesses, including shops, restaurants and pubs, had reopened. Support bubbles had been introduced, allowing for a single-adult household to interact with another household of any size.[2] International travel was permitted, following the introduction of travel corridors on 10 July 2020, which enabled passengers to travel to England from certain countries without self-isolating.[3] The UK government's 'Eat Out to Help Out' scheme, which ran from 3 to 31 August 2020, encouraged people to dine out.[4] Some social distancing restrictions remained in place, including maintaining a 2 metre distance between individuals (excluding household members or members of a support bubble), the wearing of face coverings on public transport and in shops, and limits on how many people could meet indoors and outdoors. [5–8] Whilst some people in the UK began to return to work, schools remained closed. A marked decrease in case incidence was seen during April 2020, and cases remained low until the onset of the second wave in August 2020.

Epidemics are largely driven by social mixing patterns and their quantification is useful for transmission modelling purposes, as well as assessing adherence to regulations and identifying sociodemographic factors associated with heterogeneities in contact rate.[9–11] The apparent association between social distancing restrictions and reduced case incidence indicates that a nuanced understanding of how individuals' contact patterns vary could inform behavioural interventions for the remainder of the outbreak. Previous contact studies have provided estimates for age-specific contact rates in Great Britain and the UK.[11–13] A cross-sectional survey of UK adults early on during the lockdown beginning in March found a substantial reduction in daily contact between people.[14]

We conducted a cross-sectional online survey between 28 July and 14 August 2020 to measure the mobility of people living in the UK, which locations people were frequenting, and the number of non-household contacts people were making. We aimed to quantify non-household contact behaviour and adherence to self-isolation and shielding guidance. The study period coincided with the start of the second wave of SARS-CoV-2 infection in the UK, when hospital admissions for COVID-19 were at their lowest rate since April.[15]

## METHODS

### Survey Methodology

Data collection was conducted through an anonymous online questionnaire; the study was branded the CoCoNet (COVID-19 Contact Network) survey. The survey was open to anyone living in the UK at the time of the survey. There was no lower age limit for participation, with children under 13 required to complete the survey with a parent or guardian. The inclusion

For peer review only

criteria for participants were that they completed the question on residency location and that they were resident in the UK at the time of the survey.

The survey was promoted through a university press release, engagement with the media, and posts on social media directing potential participants to the study website: https://www.lancaster.ac.uk/health-and-medicine/research/coconet-study/.

Demographic information from participants, including age, sex, ethnicity, home location (first part of postcode) and their employment or school situation, was collected. Participants were asked about their household size, as well as the formation and size of support bubbles they may belong to. Participants were asked about their activities on the previous day (the contact reporting day), including whether they left their household and the number and characteristics of non-household contacts encountered. The questionnaire is presented in Supplementary Material.

To reduce participant burden, a triage question on how many people participants had met the previous day determined the level of information collected on contacts. Participants reporting fewer than 15 contacts were asked to estimate the age of each contact they made, whether they met the contact indoors or outdoors, and if anyone from their household had also met that contact the same day. Participants who reported 15 or more contacts were asked to estimate the number of contacts made with different age groups, and whether they had met most of their contacts indoors or outdoors.

Responses recorded between 00:00BST 28 July and 18:00BST 14 August 2020 were included in the analysis. Partial responses to the survey were analysed if the first compulsory question asking which part of the UK a participant resided in was answered. If a participant exited the online survey early, we used their responses up to and including the last question they saw.

**Primary and secondary outcome measurements**

Our primary outcome was non-household contact rate. A non-household contact was defined as someone with whom the participant had a face-to-face conversation with, excluding members of their own household. A participant who remained at home could still make non-household contacts by having visitors to their home.

Secondary outcomes were whether contacts occurred indoors or outdoors, propensity to leave home over a 7 day period, ability to socially distance, locations visited, furthest distance travelled from home, and membership of support bubble.

**Descriptive analysis**

Representativeness was assessed by visual comparison of participant demographics with respective Office for National Statistics (ONS) 2019 mid-year estimates.[16,17] The mean number of non-household contacts was calculated and stratified by age, sex and household size, and was compared to reported values from other social contact surveys. Adherence to social distancing guidance was assessed by calculating the proportion of participants who left home in the past 7 days, the distribution of furthest distance travelled in the past 7 days, and the proportion of participants who felt able to maintain a recommended physical distance during contact with others. Non-responses were excluded from analyses.


For peer review only - http://bmjopen.bmj.com/site/about/guidelines.xhtml

## Predictors of contact frequency

To identify characteristics of the participant associated with their rate of daily non-household contact we fitted a negative binomial model to the daily number of non-household contacts reported by participants. Explanatory variables included in the model a priori were: age; sex; ethnicity; nation of residence (England, Northern Ireland, Scotland or Wales); household size; dwelling type; whether the contact reporting day was a weekend or week day; whether the participant had left their home on the contact reporting day; participant's working situation; participant's COVID-19 circumstance. To support our hypothesis-driven choice of model parameters, we also conducted a forward stepwise model selection process, with our previously selected explanatory variables used as candidate variables; see supplementary materials. Statistical analyses were conducted using R version 4.0.2.[18]

## Ethics Statement

This study was approved by the Faculty of Health and Medicine Ethics Committee at Lancaster University (reference FHMREC19135). Participation in the study was voluntary, with each participant (and where appropriate parent or guardian) giving their consent before proceeding.

## Patient and public involvement statement

Patients or the public were not involved in the design, or conduct, or reporting, or dissemination plans of our research. However, as the online survey was promoted via social media, members of the public were free to further promote it via social media links.

# RESULTS

## Participant demographics

We received 5,383 survey responses recorded between 28 July 2020 and 14 August 2020; 5,143 responses met our inclusion criteria.[19] Most participants were aged 40-59 (55.3%, 2813/5090); Table 1, Figure 1A. We recorded fewer responses from participants in the youngest age groups, 0-9 year olds (0.1%, 5/5090) and 10-19 year olds (0.7%, 38/5090), and in the oldest age group, aged 80+ (0.4%, 21/5090). Males, non-white ethnicities, and residents of Northern Ireland and Wales were under-represented in our sample.



For peer review only - http://bmjopen.bmj.com/site/about/guidelines.xhtml

178 **Table 1:** Participant demography and UK ONS 2019 mid-year estimates. N is the number of
179 participants who provided a response to the question.

| | Number of participants (%) | UK ONS mid-year estimates (2019)* |
|---|---|---|
| Age group (N = 5,090)† | | |
| 0-9 | 5 (0.1%) | 12.0% |
| 10-19 | 38 (0.7%) | 11.4% |
| 20-29 | 256 (5.0%) | 13.0% |
| 30-39 | 598 (11.7%) | 13.3% |
| 40-49 | 1183 (23.2%) | 12.6% |
| 50-59 | 1630 (32.0%) | 13.6% |
| 60-69 | 1065 (20.9%) | 10.7% |
| 70-79 | 294 (5.8%) | 8.4% |
| 80+ | 21 (0.4%) | 5.0% |
| Sex (N = 5,090)† | | |
| Female | 4017 (78.9%) | 50.6% |
| Male | 1051 (20.6%) | 49.4% |
| Prefer not to say | 22 (0.4%) | - |
| Ethnicity (N = 5,090) | | |
| White | 4880 (95.9%) | 86.0% |
| Mixed/Multiple ethnic groups | 49 (1.0%) | 2.2% |
| Asian/Asian British | 50 (1.0%) | 7.5% |
| Black/African/Caribbean/Black British | 11 (0.2%) | 3.3% |
| Other ethnic groups | 7(0.1%) | 1.0% |
| Prefer not to say | 16 (0.3%) | - |
| No response | 77 (1.5%) | - |
| Nation (N = 5,143)† | | |
| England | 4714 (91.7%) | 84.3% |
| Northern Ireland | 33 (0.6%) | 2.8% |
| Scotland | 254 (4.9%) | 4.7% |
| Wales | 142 (2.8%) | 8.2% |
| Household size (N = 5,073)† | | |
| 1 | 878 (17.3%) | 29.5% |
| 2 | 1911 (37.7%) | 34.5% |
| 3 | 987 (19.5%) | 15.4% |
| 4 | 907 (17.9%) | 13.9% |
| 5 | 287 (5.7%) | 4.5% |
| 6+ | 103 (2.0%) | 2.1% |

For peer review only

* Ethnicity estimates from 2011 census data.

† Question required a response from participants to progress through the online survey.

For peer review only

**Mobility**

We found 33.7% (95% confidence interval (CI) 32.4 to 35.0) of participants left their home every day over a 7-day period; Table 2. Over the same time period, most participants travelled less than 10 miles from home, but some longer-range travel (50+ miles) occurred.

For peer review only

**Table 2:** Ability of participants to social distance, membership and size of support bubbles, locations visited and mobility of participants. N is the number of participants who provided a response to the question.

| | **Number of participants (%)** |
|---|---|
| Maintaining social distance yesterday (N = 3,249)[†] | |
| All of the time | 1910 (58.8%) |
| More than half of the time | 934 (28.7%) |
| Less than half of the time | 296 (9.1%) |
| None of the time | 89 (2.7%) |
| Not sure | 20 (0.6%) |
| Part of a support bubble (N = 5,066)[†] | |
| Yes | 2029 (40.1%) |
| No | 3037 (59.9%) |
| Support bubble size (N = 2,011) | |
| 1 | 866 (43.1%) |
| 2 | 560 (27.8%) |
| 3 | 229 (11.4%) |
| 4 | 201 (10.0%) |
| 5+ | 155 (7.7%) |
| No response | 18 |
| Frequency of leaving home in past 7 days (N = 4,896) | |
| 0 days | 82 (1.7%) |
| 1 day | 281 (5.7%) |
| 2 days | 518 (10.6%) |
| 3 days | 605 (12.4%) |
| 4 days | 568 (11.6%) |
| 5 days | 650 (13.3%) |
| 6 days | 537 (11.0%) |
| 7 days | 1650 (33.7%) |
| Not sure | 5 (0.1%) |
| No response | 30 |
| Locations visited yesterday (N = 4,034) | |
| Someone's home | 615 (15.2%) |
| School or workplace | 612 (15.2%) |
| Doctor's surgery or healthcare facility | 182 (4.5%) |
| Supermarket or convenience store | 1473 (36.5%) |
| Other shops or retail spaces | 596 (14.8%) |

For peer review only

| | Number of participants (%) |
|---|---|
| Restaurant, café or pub | 553 (13.7%) |
| For a walk or exercise | 2178 (54.0%) |
| Other | 808 (20.0%) |
| No response | 0 |
| Furthest distance travelled in past 7 days (N = 4,913) | |
| Under 2 miles | 886 (18.0%) |
| 2 - 9 miles | 1682 (34.2%) |
| 10 - 19 miles | 848 (17.3%) |
| 20 - 49 miles | 669 (13.6%) |
| 50+ miles | 828 (16.9%) |
| No response | 13 |

189 † Question required a response from participants to progress through the online survey.

For peer review only

**Non-household contacts**

A total of 14,388 non-household contacts were recorded by 5,037 participants. The mean rate of non-household contacts was 2.9 $d^{-1}$ (95%CI 2.7 to 3.0). This is a notably lower rate of non-household contact than recorded from pre-pandemic surveys; Table S2. We found 33.4% (95%CI 32.1 to 34.7) of participants made no non-household contacts. The degree distribution of non-household contacts has a long right-hand tail (95th percentile: 10 contacts $d^{-1}$, maximum 130 contacts $d^{-1}$); Figure 1B. We also quantified the non-household contact rate of household members of participants; see supplementary materials.

Mean non-household contact rate varied by age and was highest among 10-19 year olds (mean 3.6, 95%CI 1.6 to 6.5); Figure 1C. We found moderate assortative mixing by age, in line with both current and pre-pandemic contact studies; Figure S1A. We found that the mean daily non-household contact rate by participant age group was substantially lower when compared to pre-pandemic POLYMOD study; see supplementary materials, Figure S1B. A notable decrease in contact rate was found between people aged under 60 mixing with others aged under 60, with the largest reduction in contact rate seen across all age groups when mixing with 0-19 year olds; Figure S1B.

**Participant characteristics and non-household contact rate**

We identified the association of participant characteristics with the rate of non-household contact using a multiple regression model; Figure 2, Table S3. We found no association of non-household contact rate with sex or day of the week. Contact rate varied by participant age: participants aged 30-39 (adjusted incidence rate ratio (aIRR) 0.86, 95% CI 0.76 to 0.97), aged 40-49 (aIRR 0.90, 95%CI 0.82 to 1.00) and those aged 60-69 (aIRR 0.89, 95%CI 0.79 to 1.00) reported a lower rate of contact than participants aged 50-59. We found that Asian and Asian British participants had a lower rate of contact than White participants (aIRR 0.54, 95%CI 0.36 to 0.82). Participants residing in Scotland had a lower contact rate than those living in England (aIRR 0.80, 95%CI 0.68 to 0.95), whereas participants in Wales had a higher contact rate (aIRR 1.22, 95%CI 0.99 to 1.50).

Leaving home was associated with a higher non-household contact rate than staying at home (aIRR 5.58, 95%CI to 4.92 to 6.33). Attending a workplace (aIRR 3.33, 95%CI 3.02 to 3.66), being self-employed (aIRR 1.63, 95%CI 1.43 to 1.87) or working in healthcare (aIRR 5.10, 95%CI 4.29 to 6.10) was associated with a significantly higher rate of non-household contact than working at home.

**Social distancing characteristics of shielding and self-isolating individuals**

There were 353 (6.9%, 353/5073) participants who reported their COVID circumstance to be shielding, either due to being a vulnerable individual or living with a vulnerable individual. Additionally, 136 (2.7%, 136/5073) participants reported their COVID circumstance as self-isolating. Shielding individuals tended to be older than non-shielding individuals; Table S4.

Shielding and self-isolating participants were less likely to leave their home compared to those reporting their situation to be 'not self-isolating or shielding': 58.6% (95%CI 53.2 to 63.8) of shielding individuals, 52.6% (95%CI 43.8 to 61.2) of self-isolating individuals, and 82.7% (95%CI 81.6 to 83.8) of other participants reported leaving their home during the contact day; Table S4. The majority of shielding and self-isolating participants adhered to

For peer review only

contemporary social distancing guidelines: 70.1% (95%CI 62.5 to 76.9) of shielding participants and 73.6% (95%CI 59.7 to 84.7) of self-isolating participants reported maintaining social distance at all time with contacts met the previous day; Table S4.

Shielding and self-isolating individuals made fewer contacts per day outside of the household than non-shielding or isolating individuals. The unadjusted rate of non-household contact was 1.3 $d^{-1}$ (95%CI 1.1 to 1.5) amongst shielding participants, 1.2 $d^{-1}$ (95%CI 0.7 to 2.1) for self-isolating participants and 3.1 $d^{-1}$ (95%CI 2.9 to 3.2) for participants who were not self-isolating or shielding. After adjusting for other variables, we found vulnerable individuals shielding had a marginally lower non-household contact rate than those not shielding or self-isolating (aIRR 0.82, 95%CI 0.66 to 1.01). Those self-isolating as a precaution or under Test and Trace instructions had a lower non-household contact rate than individuals not shielding or self-isolating (aIRR 0.58, 95%CI 0.43 to 0.79); Figure 2. Individuals who reported as self-isolating with symptoms had a higher rate of non-household contact than those not self-isolating or shielding (aIRR 4.05, 95%CI 1.94 to 9.72). However, a single participant in this group reported a very large number of contacts on their contact day. This is not necessarily an example of non-adherence to social distancing guidance, as contact day and current day are different days. Our questionnaire design asked about contact on the day prior to completing the survey, which would be the day of their current COVID-19 situation. When we exclude this individual from our analysis we found no significant difference in contact rate; see Table S5.

**Ability to maintain social distancing**

Participants were asked how much of the time they were able to maintain social distance from everyone they had met the previous day, excluding members of their household and support bubble. We found 58.8% (95%CI 57.1 to 60.5) of participants felt able to maintain social distancing at all times, while 2.7% (95%CI 2.2 to 3.4) felt unable to maintain social distance at any time. We found that age and employment situation were associated with being able to 'maintain social distance more than half of the time'; Table S6. Participants aged 30-39 felt less able to maintain social distance more than half of the time compared to 50-59 year olds (adjusted odds ratio (aOR) 0.66, 95%CI 0.46 to 0.95). Healthcare professionals (aOR 0.26, 95%CI 0.17 to 0.40) and those attending their workplace in-person (aOR 0.71, 95%CI 0.53 to 0.96) were less likely to be able to maintain social distance than those working from home.

**Location of encounters**

Transmission risk of SARS-CoV-2 is thought to be greater in enclosed, non-ventilated spaces and lower in outdoor environments.[20] To assess how interactions may be distributed by these settings, we asked participants reporting fewer than 15 individual contacts whether each contact was made indoors or outdoors, and asked all participants if they met all or the majority of contacts indoors or outdoors. The distribution of contacts by indoor/outdoor setting was bimodal: nearly half of participants reported meeting all of their non-household contacts indoors (48.8%, 95%CI 47.0 to 50.6), while 33.7% (95%CI 32.1 to 35.4) of participants reported meeting all of their non-household contacts outdoors. We also explored the non-household contacts of participants that remained at home (visitors); see supplementary materials.



For peer review only

## DISCUSSION

We found the daily rate of social contact was considerably lower than that measured prior to 2020, despite our study period corresponding to a time when the COVID-19 pandemic social distancing restrictions were at their most relaxed during 2020 in the UK.[11–13,21] The Comix study of UK social contact rates reported a greatly reduced rate in March 2020 which increased during summer 2020, with the highest rate of contact recorded in August remaining markedly lower than pre-pandemic contact rate estimates.[22] A similar increase in contact rate following lockdown was observed by Latsuzbaia *et al.* in Luxembourg.[23]

Contact rates and ability to follow social distancing guidelines was associated with age and occupation. The older age groups (70-79, 80+), those at highest risk of severe COVID-19 outcomes, had the lowest non-household contact rates, and they mixed most often with 20-59 year olds. Individuals attending a workplace, or those self-employed or working in healthcare, had a higher daily non-household contact rate than those working from home, representing additional potential infection risk. A small proportion of participants reported making a large number (more than 50) of non-household contacts; these were exclusively participants who reported their employment situation as either attending their workplace in-person or working as a healthcare professional. Although the UK government was encouraging people to return to work at this time, we found that a high proportion of employed individuals (70.0%, excluding healthcare workers and those self-employed) continued to work from home.[24] In contrast to pre-pandemic contacts surveys, we found no association between non-household contact rate and day of the week.[11,13]

Black and Asian individuals have been shown to be at increased risk of SARS-CoV-2 infection in comparison to White individuals, possibly due to larger households, being more likely to be employed as essential workers, and less able to work from home.[25,26] However, after accounting for home-working, we found that individuals of Asian and Asian British ethnicity had a significantly lower non-household contact rate than White participants. This suggests that workplaces may be more dominant as a source of infection for these individuals than previously thought. [27]

The majority of participants reported being able to maintain social distance from others more than half of the time and very few participants reported failing to maintaining social distance at all, a similar observation made in a UK behavioural cohort.[28] Healthcare professionals and employees attending their workplace in-person were less able to maintain physical distance from people they encountered than people working from home. This highlights the increased risk of infection that some workers may face; occupations which require employees to interact closely with a large number of people are associated with an increased likelihood of exposure to COVID-19 and clusters of cases developing at a workplace.[29–31]

We found some evidence of non-adherence to self-isolating and shielding guidelines, with a high proportion of self-isolating and shielding participants leaving their home the previous day. Smith *et al.* also found low adherence to isolation instructions among the UK population during March through August 2020.[32] We found that a large proportion of self-isolating and shielding participants (including those living with vulnerable individuals) made non-


For peer review only - http://bmjopen.bmj.com/site/about/guidelines.xhtml

household contacts, suggesting shielding and self-isolating individuals needed greater support to further reduce their number of interactions and to minimise infection risk.

Participants who were self-isolating as a precautionary measure, or after having been contacted by Test and Trace, reported fewer contacts than those not shielding or self-isolating. However, participants self-isolating due to experiencing symptoms or when a member of their household had symptoms did not have reduced contact rate, possibly due to the small number of participants reporting these circumstances. Participants who reported 'not sure' as their COVID circumstance had a significantly lower non-household contact rate than those not self-isolating or shielding. This may have been due to a pause in shielding guidance coinciding with the release of the survey, which may have left participants unsure of their current circumstance.[33–35]

This survey captured the point in time where cases were starting to consistently rise for the first time since March 2020, with the reproduction number estimated to be between 0.8 and 1.1.[15,36–38] The level of social mixing in the UK at the time of this survey enabled epidemic growth.

This study was likely subject to recruitment bias, as the survey was online and open to anyone living in the UK with no active recruitment process. The survey was under-represented by children, teenagers, young adults and the very elderly, as well as ethnic minorities. In particular, underrepresentation of the very elderly (80+) limited our ability to gain insight into mixing patterns of the age group at highest risk of severe COVID-19 disease. Additionally, as we asked participants to report their contact rate, the study may have suffered from recall bias. If a participant reported meeting 15 or more contacts, information was asked about their contacts collectively rather than as individual interactions. When grouping contacts into age groups, participants could select up to '20+' contacts for each age group, which may have led to us underestimating some participant's non-household contact rates; see supplementary materials. Participants were asked about their current COVID circumstance and contact behaviour for consecutive days (contacts were those made the previous day), which may bias the association of contact rate with COVID circumstance.


For peer review only - http://bmjopen.bmj.com/site/about/guidelines.xhtml

**Acknowledgements**
We would like to thank the participants of the study for providing their time and information, and Prof Julia Gog OBE and Rev Richard Coles for helping to promote the survey.

**Contributions**
JREB, CPJ and JMR all conceived and designed the study. JREB conducted the analysis and wrote the first draft of the manuscript. All authors edited the manuscript.

**Data Availability Statement**
Data are available in a public, open access repository. The survey data are available from Lancaster University's research directory at: https://doi.org/10.17635/lancaster/researchdata/476. License: Creative Commons Attribution licence (CC BY).

**Competing Interests**
We declare we have no competing interests.

**Funding**
JREB is supported by a Lancaster University Faculty of Health and Medicine doctoral scholarship. JMR and CPJ were supported by UKRI through the JUNIPER modelling consortium [grant number MR/V038613/1].

For peer review only

## References


1 Cabinet Office. Staying at home and away from others (social distancing). Cabinet Office. 2020.https://www.gov.uk/government/publications/full-guidance-on-staying-at-home-and-away-from-others/full-guidance-on-staying-at-home-and-away-from-others (accessed Aug 2020).

2 Department of Health and Social Care. Making a support bubble with another household. 2020.https://www.gov.uk/guidance/making-a-support-bubble-with-another-household (accessed Sep 2020).

3 Department for Transport. Coronavirus (COVID-19): travel corridors. 2020.https://www.gov.uk/guidance/coronavirus-covid-19-travel-corridors (accessed Sep 2020).

4 GOV UK. Register your establishment for the Eat Out to Help Out Scheme. GOV.UK. 2020.https://www.gov.uk/guidance/register-your-establishment-for-the-eat-out-to-help-out-scheme (accessed Feb 2021).

5 Cabinet Office. Staying alert and safe (social distancing). 2020.https://www.gov.uk/government/publications/staying-alert-and-safe-social-distancing (accessed Aug 2020).

6 NI direct government services. Coronavirus (COVID-19). 2020.https://www.nidirect.gov.uk/campaigns/coronavirus-covid-19 (accessed Sep 2020).

7 Scottish government. Coronavirus (COVID-19) Phase 3: Scotland's route map update. 2020.https://www.gov.scot/publications/coronavirus-covid-19-framework-decision-making-scotlands-route-map-through-out-crisis-phase-3-update/pages/8/ (accessed Sep 2020).

8 Welsh Government. Coronavirus (COVID-19) social distancing guidance for everyone in Wales. 2020.https://gov.wales/coronavirus-social-distancing-guidance (accessed Sep 2020).

9 Wallinga J, Teunis P, Kretzschmar M. Using data on social contacts to estimate age-specific transmission parameters for respiratory-spread infectious agents. *Am J Epidemiol* 2006;**164**:936–44. doi:10.1093/aje/kwj317.

10 Read JM, Edmunds WJ, Riley S, *et al.* Close encounters of the infectious kind: methods to measure social mixing behaviour. *Epidemiol Infect* 2012;**140**:2117–30. doi:10.1017/S0950268812000842.

11 Mossong J, Hens N, Jit M, *et al.* Social contacts and mixing patterns relevant to the spread of infectious diseases. *PLoS Med* 2008;**5**:e74. doi:10.1371/journal.pmed.0050074.

12 Danon L, Read JM, House TA, *et al.* Social encounter networks: characterizing Great Britain. *Proc Biol Sci* 2013;**280**:20131037. doi:10.1098/rspb.2013.1037.

13 Klepac P, Kucharski AJ, Conlan AJK, *et al.* Contacts in context: large-scale setting-specific social mixing matrices from the BBC Pandemic project. Epidemiology. 2020.

14 Jarvis CI, Van Zandvoort K, Gimma A, *et al.* Quantifying the impact of physical distance measures on the transmission of COVID-19 in the UK. *BMC Med* 2020;**18**:124. doi:10.1186/s12916-020-01597-8.


For peer review only

15 Public Health England. Coronavirus (COVID-19) in the UK. 2020.https://coronavirus.data.gov.uk (accessed Aug 2020).

16 Office for National Statistics. Estimates of the population for the UK, England and Wales, Scotland and Northern Ireland, 2020. 2020.https://www.ons.gov.uk/peoplepopulationandcommunity/populationandmigration/populationestimates/datasets/populationestimatesforukenglandandwalesscotlandandnorthernireland (accessed Aug 2020).

17 Office for National Statistics. Families and households. 2019.https://www.ons.gov.uk/peoplepopulationandcommunity/birthsdeathsandmarriages/families/datasets/familiesandhouseholdsfamiliesandhouseholds (accessed Aug 2020).

18 The R Project for Statistical Computing. https://www.r-project.org/ (accessed 19 Aug 2020).

19 Bridgen J, Jewell C, Read J. CoCoNet manuscript data. 2021. doi:10.17635/LANCASTER/RESEARCHDATA/476.

20 Nishiura H, Oshitani H, Kobayashi T, *et al.* Closed environments facilitate secondary transmission of coronavirus disease 2019 (COVID-19). *MedRxiv* Published Online First: 2020.https://www.medrxiv.org/content/10.1101/2020.02.28.20029272v1.abstract.

21 Eames KTD, Tilston NL, Brooks-Pollock E, *et al.* Measured dynamic social contact patterns explain the spread of H1N1v influenza. *PLoS Comput Biol* 2012;**8**:e1002425. doi:10.1371/journal.pcbi.1002425.

22 Jarvis CI, Gimma A, van Zandvoort K, *et al.* The impact of local and national restrictions in response to COVID-19 on social contacts in England: a longitudinal natural experiment. *BMC Med* 2021;**19**:52. doi:10.1186/s12916-021-01924-7.

23 Latsuzbaia A, Herold M, Bertemes J-P, *et al.* Evolving social contact patterns during the COVID-19 crisis in Luxembourg. *PLoS One* 2020;**15**:e0237128. doi:10.1371/journal.pone.0237128.

24 GOV UK. Prime Minister’s statement on coronavirus (COVID-19): 17 July 2020. 2020.https://www.gov.uk/government/speeches/pm-statement-on-coronavirus-17-july-2020 (accessed 2021).

25 Sze S, Pan D, Nevill CR, *et al.* Ethnicity and clinical outcomes in COVID-19: A systematic review and meta-analysis. *EClinicalMedicine* 2020;**29**:100630. doi:10.1016/j.eclinm.2020.100630.

26 Public Health England. Disparities in the risk and outcomes of COVID-19. 2020.

27 Office for National Statistics. Updating ethnic contrasts in deaths involving the coronavirus (COVID-19), England and Wales: deaths occurring 2 March to 28 July 2020. ONS. 2020.https://www.ons.gov.uk/peoplepopulationandcommunity/birthsdeathsandmarriages/deaths/articles/updatingethniccontrastsindeathsinvolvingthecoronaviruscovid19englandandwales/latest (accessed Jan 2021).

28 Fancourt D, Bu F, Mak HW, *et al.* Covid-19 Social Study Results Release 25. *UCL* 2020.

29 Office for National Statistics. Which occupations have the highest potential exposure to the coronavirus (COVID-19)? 2020.https://www.ons.gov.uk/employmentandlabourmarket/peopleinwork/employmentande

For peer review only

mployeetypes/articles/whichoccupationshavethehighestpotentialexposuretothecoronavirusco vid19/2020-05-11 (accessed Aug 2020).

30 European Centre for Disease Prevention and Control. COVID-19 clusters and outbreaks in occupational settings in the EU/EEA and the UK. 2020.https://www.ecdc.europa.eu/en/publications-data/covid-19-clusters-and-outbreaks-occupational-settings-eueea-and-uk (accessed 2021).

31 Industrial Injuries Advisory Council. COVID-19 and occupation: position paper 48. 2021.

32 Smith LE, Potts HWW, Amlot R, *et al.* Adherence to the test, trace and isolate system: results from a time series of 21 nationally representative surveys in the UK (the COVID-19 Rapid Survey of Adherence to Interventions and Responses [CORSAIR] study). *MedRxriv* Published Online First: 2020. doi:10.1101/2020.09.15.20191957.

33 Department of Health and Social Care. Updates to the shielding programme on national and regional levels. 2020.https://www.gov.uk/government/news/updates-to-the-shielding-programme-on-national-and-regional-levels (accessed Sep 2020).

34 NI direct government services. Coronavirus (COVID-19): pausing of shielding for extremely vulnerable people. 2020.https://www.nidirect.gov.uk/articles/coronavirus-covid-19-pausing-shielding-extremely-vulnerable-people (accessed Sep 2020).

35 Scottish government. Shielding to be paused. 2020.https://www.gov.scot/news/shielding-to-be-paused/ (accessed Sep 2020).

36 Children's Task and Finish Group. Update on Children, Schools and Transmission. 2020.https://www.gov.uk/government/publications/tfc-children-and-transmission-4-november-2020 (accessed May 2021).

37 Scientific Advisory Group for Emergencies. SPI-M-O: Consensus statement on COVID-19, 12 August 2020. GOV.UK. 2020.https://www.gov.uk/government/publications/spi-m-o-consensus-statement-on-covid-19-12-august-2020 (accessed Jun 2021).

38 Scientific Advisory Group for Emergencies. SPI-M-O Consensus Statement on COVID-19, 19 August 2020. GOV.UK. 2020.https://assets.publishing.service.gov.uk/government/uploads/system/uploads/attachment_data/file/916883/spi-m-o-consensus-statement-s0702-sage-52-200819.pdf (accessed Jun 2021).

For peer review only

**Figure Legends**

**Figure 1.** (A) Age and sex distribution of participants with ONS 2019 mid-year estimates. (B) Degree distribution of non-zero contacts. (C) Distribution of reported non-zero contact rate by age group. Note, log scale of x-axis in B and C.

**Figure 2.** Adjusted incidence rate ratios for number of non-household contacts reported for selected variables.

For peer review only

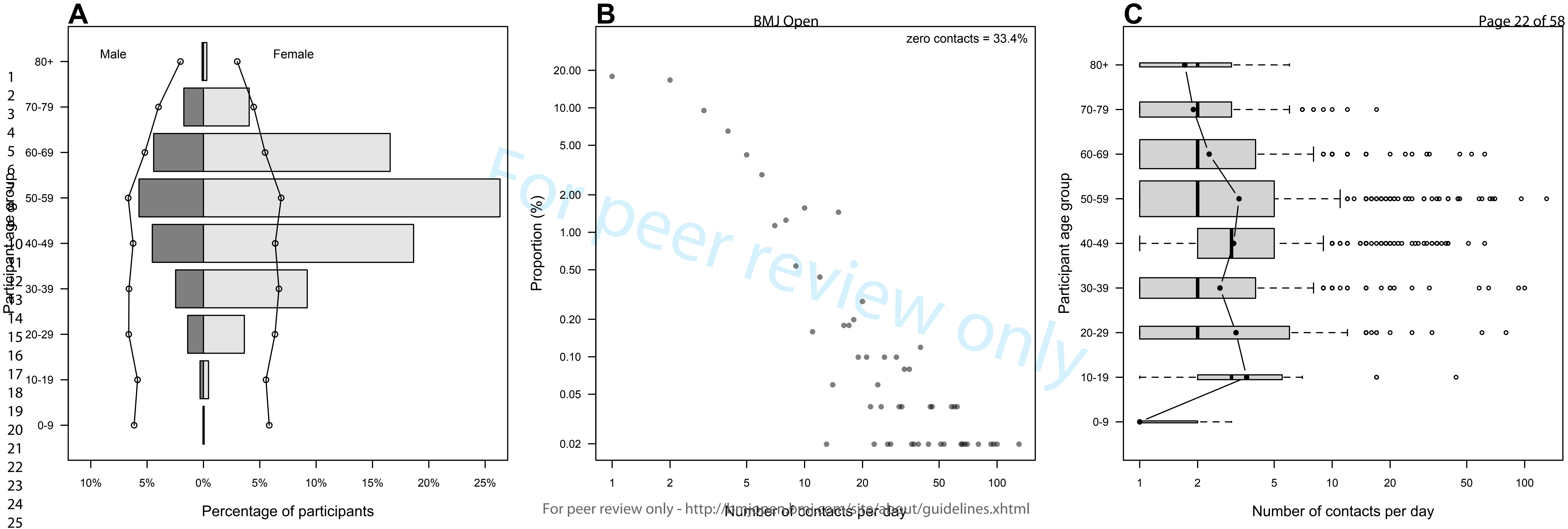


For peer review only

For peer review only - http://bmjopen.bmj.com/site/about/guidelines.xhtml

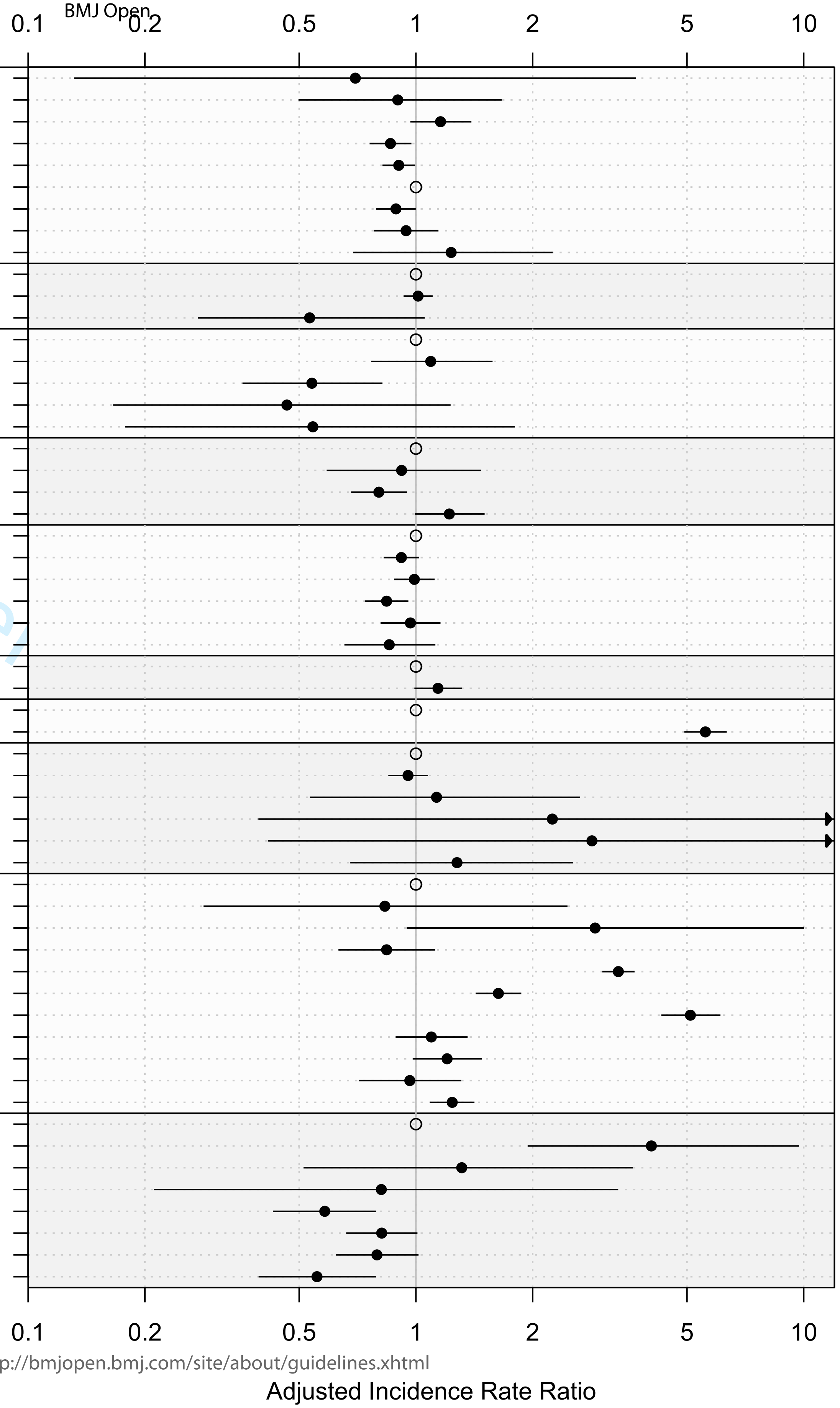


| Variable | Category | aIRR | 95%CI | p | n |
|---|---|---|---|---|---|
| Age | 0-9 | 0.70 | 0.13-3.69 | 0.673 | 5 |
| | 10-19 | 0.90 | 0.50-1.67 | 0.724 | 35 |
| | 20-29 | 1.16 | 0.97-1.39 | 0.109 | 242 |
| | 30-39 | 0.86 | 0.76-0.97 | 0.016 | 550 |
| | 40-49 | 0.90 | 0.82-1.00 | 0.039 | 1067 |
| | 50-59 | 1.00 | - | - | 1485 |
| | 60-69 | 0.89 | 0.79-1.00 | 0.046 | 996 |
| | 70-79 | 0.94 | 0.78-1.14 | 0.542 | 280 |
| | 80+ | 1.23 | 0.69-2.25 | 0.458 | 21 |
| Sex | Female | 1.00 | - | - | 3665 |
| | Male | 1.01 | 0.93-1.10 | 0.772 | 999 |
| | Prefer not to say | 0.53 | 0.27-1.05 | 0.063 | 17 |
| Ethnicity | White | 1.00 | - | - | 4569 |
| | Mixed/Multiple ethnic groups | 1.09 | 0.77-1.58 | 0.625 | 48 |
| | Asian/Asian British | 0.54 | 0.36-0.82 | 0.004 | 47 |
| | Black/African/Caribbean/Black British | 0.46 | 0.17-1.23 | 0.136 | 11 |
| | Other ethnic group | 0.54 | 0.18-1.80 | 0.308 | 6 |
| Nation | England | 1.00 | - | - | 4291 |
| | Northern Ireland | 0.92 | 0.59-1.47 | 0.713 | 27 |
| | Scotland | 0.80 | 0.68-0.95 | 0.009 | 233 |
| | Wales | 1.22 | 0.99-1.50 | 0.060 | 130 |
| Household size | 1 | 1.00 | - | - | 833 |
| | 2 | 0.92 | 0.83-1.02 | 0.105 | 1797 |
| | 3 | 0.99 | 0.88-1.12 | 0.878 | 904 |
| | 4 | 0.84 | 0.74-0.96 | 0.008 | 807 |
| | 5 | 0.97 | 0.81-1.16 | 0.717 | 250 |
| | 6+ | 0.85 | 0.65-1.12 | 0.247 | 90 |
| Day of the week | Weekday | 1.00 | - | - | 4369 |
| | Weekend | 1.14 | 0.99-1.31 | 0.066 | 312 |
| Left home | No | 1.00 | - | - | 928 |
| | Yes | 5.58 | 4.92-6.33 | <0.001 | 3753 |
| Dwelling type | House or bungalow | 1.00 | - | - | 4074 |
| | Flat, maisonette or apartment | 0.95 | 0.85-1.07 | 0.435 | 583 |
| | Mobile or temporary structure | 1.13 | 0.53-2.65 | 0.760 | 8 |
| | Assisted living facility | 2.25 | 0.39-37.68 | 0.442 | 1 |
| | Care home | 2.84 | 0.42-50.22 | 0.345 | 1 |
| | Other | 1.28 | 0.68-2.54 | 0.461 | 14 |
| School or work situation | Employed - working from home | 1.00 | - | - | 1757 |
| | School pupil - studying at home | 0.83 | 0.28-2.46 | 0.739 | 14 |
| | School pupil - attending school | 2.90 | 0.95-10.02 | 0.080 | 5 |
| | College or University student | 0.84 | 0.63-1.12 | 0.229 | 111 |
| | Employed - going to place of work | 3.33 | 3.02-3.66 | <0.001 | 836 |
| | Self Employed | 1.63 | 1.43-1.87 | <0.001 | 390 |
| | Healthcare professional | 5.10 | 4.29-6.10 | <0.001 | 154 |
| | Unemployed | 1.10 | 0.89-1.36 | 0.399 | 149 |
| | Furloughed | 1.20 | 0.98-1.48 | 0.074 | 157 |
| | Unable to work | 0.96 | 0.71-1.31 | 0.815 | 89 |
| | Retired | 1.24 | 1.09-1.42 | 0.001 | 1019 |
| COVID circumstance | Not self isolating or shielding | 1.00 | - | - | 4170 |
| | Self isolating - I have symptoms | 4.05 | 1.94-9.72 | 0.001 | 7 |
| | Self isolating - Someone in my household has symptoms | 1.31 | 0.51-3.63 | 0.604 | 7 |
| | Self isolating - Someone in my support bubble has symptoms | 0.81 | 0.21-3.32 | 0.771 | 4 |
| | Self isolating - precaution/told to by Test and Trace | 0.58 | 0.43-0.79 | <0.001 | 110 |
| | Shielding - I am a vulnerable individual | 0.82 | 0.66-1.01 | 0.056 | 197 |
| | Shielding - I live with a vulnerable individual | 0.79 | 0.62-1.02 | 0.065 | 123 |
| | Not sure | 0.56 | 0.39-0.79 | 0.001 | 63 |

# Supplementary Material: Social mixing patterns in the UK following the relaxation of COVID-19 pandemic restrictions: a cross-sectional online survey

## Contents

*Analysis* ... *2*
Age-specific mixing rates ... 2
Variables associated with variation in non-household contact rate - model selection ... 2
Contact clustering or transitivity ... 2
Visiting other households ... 3
Household visits ... 3
Support bubbles ... 3
Survey methodology - limitations ... 3
*Figures* ... *4*
*Tables* ... *5*
*References* ... *19*
*Survey Questions* ... *20*

For peer review only

# Analysis

## Age-specific mixing rates

To calculate age-specific mixing patterns we first defined matrix $C_{ij}$, where $C_{ij}$ was the number of non-household contacts reported between participant age groups *i* and contact age groups *j*. The mean contact rate per age group ($M_{ij}$) was given by

$$M_{ij} = \frac{C_{ij}}{N_i}$$

where $N_i$ was the number of participants in age group *i*. As a measure of uncertainty, we calculated confidence intervals by taking 1,000 bootstrapped samples of participants.

Similarly, age-specific non-household contact rates were derived from the POLYMOD data.[1] We calculated the percentage decrease of age-specific non-household contact rates between the POLYMOD data and the CoCoNet data; Figure S1.

We found moderate assortative mixing by age, in line with both current and pre-pandemic contact studies (q = 0.38, 95%CI 0.17 to 0.58); Figure S1. Of all ages under 80 years old, 30-39 year olds had the highest non-household contact rate with those aged 80 or over; 4.8% (95%CIs 3.77 to 6.04) of non-household contacts reported by 30-39 years olds were with someone aged 80+.

## Variables associated with variation in non-household contact rate - model selection

To identify characteristics of the participant associated with their rate of daily non-household contact we fitted a negative binomial model to the daily number of non-household contacts reported by participants. We then used our selected explanatory variables as candidate variables for a forward stepwise model selection process.

Participant age group, sex, ethnicity, working situation and COVID circumstance were included as explanatory variables in all models. Candidate explanatory variables were: nation of residence (England, Northern Ireland, Scotland or Wales); household size; dwelling type; whether the contact reporting day was a weekend or week day; whether the participant had left their home on the contact reporting day.

The model selection process selected the following variables from candidate variables: age, sex, ethnicity, working situation, COVID circumstance, nation, household size, day of the week, whether the participant had left their home as explanatory variables. Dwelling type was not selected as an explanatory variable. Their association with the rate of non-household contact in the fully adjusted model is shown in Table S7.

## Contact clustering or transitivity

Participants who made fewer than 15 contacts and did not live alone were asked if anyone in their household had met each of their contacts that same day, as a measure of clustering (also called transitivity) within social networks.[2] We estimated 40.4% (95%CI 39.1 to 41.6) of non-household contacts were also met by another household member on the same day. The proportion of contacts encountered by participant and household members was highest if the contact was under 20 years old: contact aged 0 to 4 (77.5%, 95%CI 66.8 to 86.1); aged 5


For peer review only - http://bmjopen.bmj.com/site/about/guidelines.xhtml

to 9 (80.0%, 95%CI 72.7 to 86.1); aged 10 to 19 (63.2%, 95%CI 56.1 to 69.9). This indicates that non-household interactions with children tend to be made with multiple individuals from those households.

**Visiting other households**

Evidence suggests that transmission of COVID-19 often occurs within households.[3] We found that 12.2% (95%CI 11.3 to 13.1) of participants reported visiting another household. Females (aOR 1.2, 95%CI 0.98 to 1.56) and members of support bubbles (aOR 1.92, 95%CI 1.61 to 2.28) were more likely to have visited another household; Table S8.

**Household visits**

The mean rate of contacts made with non-household members (non-household contacts) by those not leaving their home was 0.4 $d^{-1}$ (95%CI 0.4 to 0.5), with 23.8% (95%CI 21.2 to 26.6) meeting one or more non-household contacts. This contact rate was significantly lower than for those who did leave their home (incidence rate ratio (IRR) 0.12, 95%CI 0.11 to 0.14, p-value <0.001).

**Support bubbles**

A substantial proportion (40.1%, 95%CI 38.7 to 41.4) of participants reported being part of a support bubble, with 43.1% (95%CI 40.9 to 45.3) joining with a single-person household. Males were less likely to be part of a support bubble (aOR 0.68, 95%CI 0.58 to 0.78); support bubble membership was not associated with age group or ethnicity. Support bubbles had a median (non-participant side) size of 2.0 (25th percentile 1.0, 75th percentile 3.0) and mean size of 2.2, (95%CI 2.1 to 2.2), and were mostly encountered two or fewer days in the past week.

**Survey methodology - limitations**

When calculating the mean number of daily non-household contacts, an assumption for the maximum number of contacts was made. The survey asked participants how many contacts they had made yesterday, with the option of '0 to 15 or more'. If participants selected '15 or more', they were asked to group the contacts they had made by age, by selecting an integer between 0 and 19 or '20+' for each contact age category. To calculate the mean number of contacts, where '20+' contacts was selected, this was assumed to be 20 contacts. We may, therefore, have underestimated the maximum number of contacts of some participants and non-household contact rates.



For peer review only - http://bmjopen.bmj.com/site/about/guidelines.xhtml

## Figures

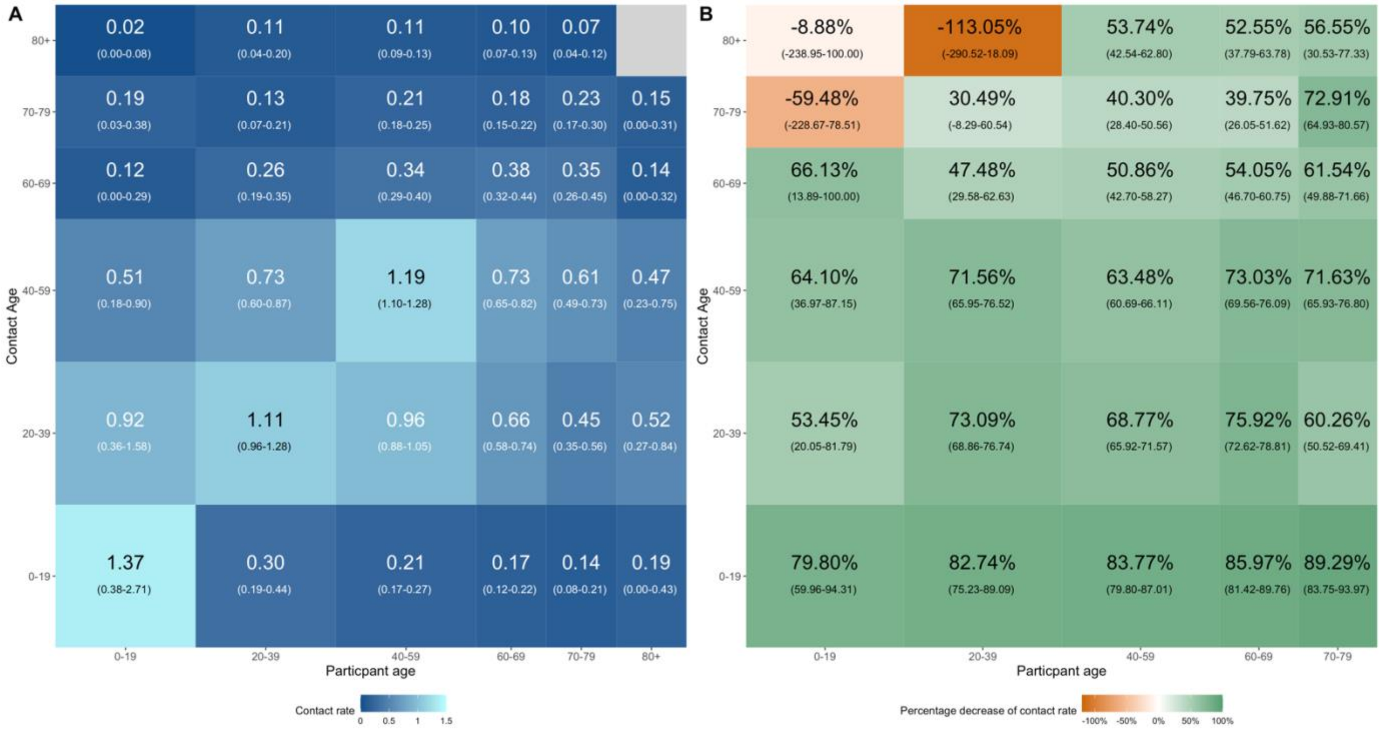


**Figure S1.** (A) Mean non-household contact rate (number of contacts per day) with different age groups reported by participant age group; bootstrapped 95% confidence intervals shown in parentheses. (B) Percentage decrease of non-household contact rate between the POLYMOD data (2005-2006) and the CoCoNet data; bootstrapped 95% confidence intervals shown in parentheses.



For peer review only - http://bmjopen.bmj.com/site/about/guidelines.xhtml

## Tables

**Table S2.** Comparison of non-household contact rates across different UK contact surveys.

| | Mean contact rate, $d^{-1}$ (25th and 75th percentile) | | | | |
|---|---|---|---|---|---|
| Study, contact type and geography of sample | **CoCoNet Number of participants** | **CoCoNet non-household contacts UK** | **CoMix non-household contacts UK** [4] | **Social Contact Survey non-household contacts, Great Britain** [5] | **POLYMOD, non-household contacts, Great Britain** [1] |
| Sampling period | | 28 July 2020 to 14 August 2020 Non-lockdown period | 24 March 2020 to 27 March 2020 Lockdown period | 2009 Pre-pandemic | 2005-2006 Pre-pandemic |
| All participants | 5,037 | 2.9 (0, 3) | 1.4 (0, 1) | 25.9 (5, 23) | 9.6 (4, 13) |
| Age group | | | | | |
| 0-9 | 5 | 1.0 (0, 1) | - | 29.8 (7, 46) | 8.9 (3, 13) |
| 10-19 | 37 | 3.6 (0, 3) | - | 43.1 (8, 40) | 12.3 (5, 18) |
| 20-29 | 250 | 3.2 (0, 3) | 1.1 (0, 1)* | 29.0 (7, 27) | 9.7 (5, 13) |
| 30-39 | 594 | 2.6 (0, 3) | 1.4 (0, 1) | 25.4 (6, 25) | 8.9 (4, 12) |
| 40-49 | 1167 | 3.1 (0, 4) | 1.4 (0, 1) | 30.8 (6, 29) | 9.8 (4, 13) |
| 50-59 | 1617 | 3.3 (0, 3) | 1.6 (0, 2) | 28.6 (6, 26) | 8.2 (3, 11) |
| 60-69 | 1056 | 2.3 (0, 3) | 1.4 (0, 2) | 23.0 (4, 18) | 8.2 (4, 11) |
| 70-79 | 290 | 1.9 (0, 3) | 1.1 (1, 1)** | 19.1 (3, 17) | 6.8 (3, 11) |
| 80+ | 21 | 1.7 (0, 2) | - | 13.2 (1,10) | - |
| Sex† | | | | | |
| Female | 3978 | 2.8 (0, 3) | 1.4 (0, 1) | 27.5 (5, 26) | 10.2 (4, 14) |
| Male | 1037 | 2.9 (0, 3) | 1.3 (0, 1) | 22.6 (4, 19) | 9.0 (4, 13) |
| Prefer not to say | 22 | 1.5 (0, 2) | - | - | - |
| Household size | | | | | |
| 1 | 875 | 2.8 (0, 3) | 1.6 (1, 2) | 24.3 (4, 20) | 7.5 (3, 11) |
| 2 | 1902 | 2.6 (0, 3) | 1.5 (0, 2) | 23.7 (5, 21) | 9.2 (4, 12) |
| 3 | 979 | 3.2 (0, 3) | 1.2 (0, 1) | 24.6 (5, 25) | 9.6 (4, 14) |
| 4 | 896 | 2.7 (0, 3) | 1.4 (0, 1) | 33.4 (6, 33) | 10.0 (4, 14) |
| 5 | 284 | 3.2 (0, 4) | 1.1 (0, 1) | 30.7 (7, 30) | 10.9 (5, 15) |
| 6+ | 101 | 3.8 (0, 4) | 1.1 (0, 1) | 45.6 (8, 36) | 10.1 (5, 15) |

* CoMix age group 18-29
** CoMix age group 70+
† Comix and POLYMOD report participants' gender rather than sex



For peer review only - http://bmjopen.bmj.com/site/about/guidelines.xhtml

**Table S3.** Adjusted incidence rate ratios for number of daily non-household contacts by select variables. Intercept of 0.41 (95 %CI 0.35-0.48). Dispersion parameter of 1.07 (95%CI 1.00-1.14)

| | **Multivariable analysis[1]** | |
|---|---|---|
| | **aIRR (95%CI)** | **p-value** |
| Age | | |
| 0-9 | 0.70 (0.13-3.69) | 0.673 |
| 10-19 | 0.90 (0.50-1.67) | 0.724 |
| 20-29 | 1.16 (0.97-1.39) | 0.109 |
| 30-39 | 0.86 (0.76-0.97) | 0.016 |
| 40-49 | 0.90 (0.82-1.00) | 0.039 |
| 50-59 | 1.00 | - |
| 60-69 | 0.89 (0.79-1.00) | 0.046 |
| 70-79 | 0.94 (0.78-1.14) | 0.542 |
| 80+ | 1.23 (0.69-2.25) | 0.458 |
| Sex | | |
| Female | 1.00 | - |
| Male | 1.01 (0.93-1.10) | 0.772 |
| Prefer not to say | 0.53 (0.27-1.05) | 0.063 |
| Ethnicity | | |
| White | 1.00) | - |
| Mixed/Multiple ethnic groups | 1.09 (0.77-1.58) | 0.625 |
| Asian/Asian British | 0.54 (0.36-0.82) | 0.004 |
| Black/African/Caribbean/Black British | 0.46 (0.17-1.23) | 0.136 |
| Other ethnic group | 0.54 (0.18-1.80) | 0.308 |
| Nation | | |
| England | 1.00 | - |
| Northern Ireland | 0.92 (0.59-1.47) | 0.713 |
| Scotland | 0.80 (0.68-0.95) | 0.009 |
| Wales | 1.22 (0.99-1.50) | 0.060 |
| Household size | | |
| 1 | 1.00 | - |
| 2 | 0.92 (0.83-1.02) | 0.105 |



For peer review only - http://bmjopen.bmj.com/site/about/guidelines.xhtml

| | Multivariable analysis[1] | |
|---|---|---|
| | aIRR (95%CI) | p-value |
| 3 | 0.99 (0.88-1.12) | 0.878 |
| 4 | 0.84 (0.74-0.96) | 0.008 |
| 5 | 0.97 (0.81-1.16) | 0.717 |
| 6+ | 0.85 (0.65-1.12) | 0.247 |
| Day of the week (contacts recorded) | | |
| Weekday | 1.00 | - |
| Weekend | 1.14 (0.99-1.31) | 0.066 |
| Left home yesterday | | |
| No | 1.00 | - |
| Yes | 5.58 (4.92-6.33) | <0.001 |
| Dwelling type | | |
| House or bungalow | 1.00 | - |
| Flat, maisonette or apartment | 0.95 (0.85-1.07) | 0.435 |
| Mobile or temporary structure | 1.13 (0.53-2.65) | 0.760 |
| Assisted living facility | 2.25 (0.39-37.68) | 0.442 |
| Care home | 2.84 (0.42-50.22) | 0.345 |
| Other | 1.28 (0.68-2.54) | 0.461 |
| School or work situation | | |
| Employed - working from home | 1.00 | - |
| School pupil - studying at home | 0.83 (0.28-2.46) | 0.739 |
| School pupil - attending school | 2.90 (0.95-10.02) | 0.080 |
| College or University student | 0.84 (0.63-1.12) | 0.229 |
| Employed - going to place of work | 3.33 (3.02-3.66) | <0.001 |
| Self Employed | 1.63 (1.43-1.87) | <0.001 |
| Healthcare professional | 5.10 (4.29-6.10) | <0.001 |
| Unemployed | 1.10 (0.89-1.36) | 0.399 |
| Furloughed | 1.20 (0.98-1.48) | 0.074 |
| Unable to work | 0.96 (0.71-1.31) | 0.815 |
| Retired | 1.24 (1.09-1.42) | 0.001 |
| COVID-19 circumstance | | |
| Not self isolating or shielding | 1.00 | - |



For peer review only - http://bmjopen.bmj.com/site/about/guidelines.xhtml

| | Multivariable analysis[1] | |
|---|---|---|
| | aIRR (95%CI) | p-value |
| Self isolating - I have symptoms | 4.05 (1.94-9.72) | 0.001 |
| Self isolating - Someone in my household has symptoms | 1.31 (0.51-3.63) | 0.604 |
| Self isolating - Someone in my support bubble has symptoms | 0.81 (0.21-3.32) | 0.771 |
| Self isolating - precaution/told to by Test and Trace | 0.58 (0.43-0.79) | <0.001 |
| Shielding - I am a vulnerable individual | 0.82 (0.66-1.01) | 0.056 |
| Shielding - I live with a vulnerable individual | 0.79 (0.62-1.02) | 0.065 |
| Not sure | 0.56 (0.39-0.79) | 0.001 |

[1]Adjusted for age,sex, ethnicity, nation, household size, day of the week, left home, dwelling type, school or work situation and COVID-19 circumstance.

*This increased contact rate is due to one participant who was self-isolating with symptoms reporting a large number of contacts (*see results*).



For peer review only - http://bmjopen.bmj.com/site/about/guidelines.xhtml

**Table S4.** Characteristics of participants who reported 'Self isolating' or 'Shielding' as their COVID circumstance. N is the number of participants who provided a response to the question.

| | Number of self-isolating participants (%) | Number of shielding participants (%) | Number of participants not self-isolating or shielding (%) |
|---|---|---|---|
| Age group | N = 136 | N = 353 | N = 4,511 |
| 0-9 | 0 (0.0%) | 0 (0.0%) | 5 (0.1%) |
| 10-19 | 1 (0.7%) | 4 (1.1%) | 33 (0.7%) |
| 20-29 | 7 (5.1%) | 12 (3.4%) | 233 (5.2%) |
| 30-39 | 11 (8.1%) | 28 (7.9%) | 549 (12.2%) |
| 40-49 | 17 (12.5%) | 64 (18.1%) | 1093 24.2%) |
| 50-59 | 41 (30.1%) | 95 (26.9%) | 1465 (32.5%) |
| 60-69 | 32 (23.5%) | 104 (29.5%) | 905 (20.1%) |
| 70-79 | 22 (16.2%) | 43 (12.2%) | 215 (4.8%) |
| 80+ | 5 (3.7%) | 3 (0.8%) | 13 (0.3%) |
| No response | 0 (0.0%) | 0 (0.0%) | 0 (0.0%) |
| Sex | N = 136 | N = 353 | N = 4,511 |
| Female | 111 (81.6%) | 286 (81.0%) | 3548 (78.7%) |
| Male | 25 (18.4%) | 65 (18.4%) | 943 (20.9%) |
| Prefer not to say | 0 (0.0%) | 2 (0.6%) | 20 (0.4%) |
| No response | 0 (0.0%) | 0 (0.0%) | 0 (0.0%) |
| Ethnicity | N = 136 | N = 353 | N = 4,511 |
| White | 126 (92.6%) | 334 (94.6%) | 4336 (96.1%) |
| Mixed/Multiple ethnic groups | 5 (3.7%) | 8 (2.3%) | 36 (0.8%) |
| Asian/Asian British | 3 (2.2%) | 2 (0.6%) | 43 (1.0%) |
| Black/African/Caribbean/Black British | 0 (0.0%) | 2 (0.6%) | 9 (0.2%) |
| Other ethnic group | 1 (0.7%) | 0 (0.0%) | 6 (0.1%) |
| Prefer not to say | 0 (0.0%) | 3 (0.8%) | 13 (0.3%) |
| No response | 1 (0.7%) | 4 (1.1%) | 68 (1.5%) |
| Left home yesterday | N = 135 | N = 350 | N = 4,495 |
| No | 64 (47.4%) | 145 (41.4%) | 778 (17.3%) |
| Yes | 71 (52.6%) | 205 (58.6%) | 3717 (82.7%) |



For peer review only - http://bmjopen.bmj.com/site/about/guidelines.xhtml

| | Number of self-isolating participants (%) | Number of shielding participants (%) | Number of participants not self-isolating or shielding (%) |
|---|---|---|---|
| No response | 0 (0.0%) | 0 (0.0%) | 0 (0.0%) |
| Part of a support bubble | N = 136 | N= 352 | N = 4,505 |
| No | 87 (64.0%) | 238 (67.6%) | 2664 (59.1%) |
| Yes | 49 (36.0%) | 114 (32.4%) | 1841 (40.9%) |
| No response | 0 (0.0%) | 0 (0.0%) | 0 (0.0%) |
| Social distancing | N = 53 | N= 167 | N = 2,989 |
| Yes, all of the time | 39 (73.6%) | 117 (70.1%) | 1728 (57.8%) |
| More than half of the time | 10 (18.9%) | 36 (21.6%) | 877 (29.3%) |
| Less than half of the time | 4 (7.5%) | 11 (6.6%) | 278 (9.3%) |
| No, none of the time | 0 (0.0%) | 3 (1.8%) | 86 (2.9%) |
| Not sure | 0 (0.0%) | 0 (0.0%) | 20 (0.7%) |
| No response | 0 (0.0%) | 0 (0.0%) | 0 (0.0%) |
| | **Mean daily non-household contact rate (IQR)** | | |
| | **Sef-isolating participants** | **Shielding participants** | **Participants not shielding or self-isolating** |
| | N = 134 | N = 348 | N = 4,484 |
| All participants | 1.2 (0, 2) | 1.3 (0, 2) | 3.1 (0, 3) |

eview only

**Table S5.** Adjusted incidence rate ratios for number of daily non-household contacts by select variables. Self-isolating individual with large number of contacts removed for this analysis (see results). Intercept of 0.41 (95 %CI 0.35-0.48). Dispersion parameter of 1.07 (95%CI 1.00-1.14)

| | Multivariable analysis[1] | |
|---|---|---|
| | **aIRR (95%CI)** | **p-value** |
| Age | | |
| 0-9 | 0.69 (0.13-3.64) | 0.662 |
| 10-19 | 0.89 (0.49-1.64) | 0.691 |
| 20-29 | 1.15 (0.97-1.39) | 0.114 |
| 30-39 | 0.86 (0.76-0.98) | 0.021 |
| 40-49 | 0.91 (0.82-1.00) | 0.044 |
| 50-59 | 1.00 | - |
| 60-69 | 0.89 (0.79-1.00) | 0.047 |
| 70-79 | 0.94 (0.78-1.14) | 0.540 |
| 80+ | 1.23 (0.69-2.25) | 0.457 |
| Sex | | |
| Female | 1.00 | - |
| Male | 1.02 (0.93-1.11) | 0.730 |
| Prefer not to say | 0.53 (0.27-1.05) | 0.063 |
| Ethnicity | | |
| White | 1.00) | - |
| Mixed/Multiple ethnic groups | 1.13 (0.79-1.62) | 0.511 |
| Asian/Asian British | 0.54 (0.36-0.82) | 0.004 |
| Black/African/Caribbean/Black British | 0.47 (0.17-1.23) | 0.137 |
| Other ethnic group | 0.54 (0.18-1.79) | 0.305 |
| Nation | | |
| England | 1.00 | - |
| Northern Ireland | 0.92 (0.59-1.47) | 0.713 |
| Scotland | 0.80 (0.68-0.95) | 0.009 |
| Wales | 1.22 (1.00-1.50) | 0.059 |
| Household size | | |
| 1 | 1.00 | - |


For peer review only - http://bmjopen.bmj.com/site/about/guidelines.xhtml

| | Multivariable analysis[1] | |
|---|---|---|
| | aIRR (95%CI) | p-value |
| 2 | 0.92 (0.83-1.02) | 0.105 |
| 3 | 0.98 (0.87-1.11) | 0.805 |
| 4 | 0.84 (0.74-0.95) | 0.007 |
| 5 | 0.97 (0.81-1.15) | 0.692 |
| 6+ | 0.85 (0.65-1.12) | 0.244 |
| Day of the week (contacts recorded) | | |
| Weekday | 1.00 | - |
| Weekend | 1.14 (0.99-1.31) | 0.068 |
| Left home yesterday | | |
| No | 1.00 | - |
| Yes | 5.54 (4.89-6.29) | <0.001 |
| Dwelling type | | |
| House or bungalow | 1.00 | - |
| Flat, maisonette or apartment | 0.95 (0.85-1.07) | 0.414 |
| Mobile or temporary structure | 1.21 (0.56-2.87) | 0.647 |
| Assisted living facility | 2.25 (0.39-37.46) | 0.441 |
| Care home | 2.78 (0.41-48.81) | 0.355 |
| Other | 1.28 (0.68-2.54) | 0.460 |
| School or work situation | | |
| Employed - working from home | 1.00 | - |
| School pupil - studying at home | 0.85 (0.29-2.50) | 0.763 |
| School pupil - attending school | 2.90 (0.95-10.00) | 0.080 |
| College or University student | 0.86 (0.65-1.15) | 0.291 |
| Employed - going to place of work | 3.34 (3.04-3.68) | <0.001 |
| Self Employed | 1.64 (1.43-1.87) | <0.001 |
| Healthcare professional | 5.12 (4.31-6.12) | <0.001 |
| Unemployed | 1.10 (0.89-1.37) | 0.369 |
| Furloughed | 1.21 (0.99-1.49) | 0.063 |
| Unable to work | 1.00 (0.74-1.35) | 0.993 |
| Retired | 1.25 (1.09-1.42) | 0.001 |
| COVID-19 circumstance | | |



For peer review only - http://bmjopen.bmj.com/site/about/guidelines.xhtml

| | Multivariable analysis[1] | |
|---|---|---|
| | aIRR (95%CI) | p-value |
| Not self isolating or shielding | 1.00 | - |
| Self isolating - I have symptoms | 0.83 (0.24-2.82) | 0.775 |
| Self isolating - Someone in my household has symptoms | 1.31 (0.51-3.62) | 0.606 |
| Self isolating - Someone in my support bubble has symptoms | 0.81 (0.21-3.30) | 0.768 |
| Self isolating - precaution/told to by Test and Trace | 0.58 (0.43-0.79) | <0.001 |
| Shielding - I am a vulnerable individual | 0.81 (0.66-1.00) | 0.050 |
| Shielding - I live with a vulnerable individual | 0.79 (0.62-1.02) | 0.065 |
| Not sure | 0.56 (0.39-0.79) | 0.001 |

[1] Adjusted for age,sex, ethnicity, nation, household size, day of the week, left home, dwelling type, school or work situation and COVID-19 circumstance.

For peer review only

**Table S6.** Association of participant characteristics and maintaining social distancing more than half of the time with contacts (adjusted odds ratios). N = 3058. Model intercept of 9.34 (7.24-12.20).

| | Adjusted Odds Ratio (95%CI)[1] | p-value |
|---|---|---|
| Age | | |
| 10-19 | 0.30 (0.08-1.25) | 0.077 |
| 20-29 | 0.62 (0.38-1.06) | 0.069 |
| 30-39 | 0.66 (0.46-0.95) | 0.024 |
| 40-49 | 0.88 (0.65-1.19) | 0.415 |
| 50-59 | 1.00 | - |
| 60-69 | 0.88 (0.61-1.28) | 0.496 |
| 70-79 | 0.91 (0.49 -1.79) | 0.778 |
| 80+ | 0.28 (0.08-1.30) | 0.064 |
| School or work situation | | |
| Employed - working from home | 1.00 | - |
| College or University student | 0.68 (0.33-1.55) | 0.335 |
| Employed - going to place of work | 0.71 (0.53-0.96) | 0.025 |
| Self Employed | 1.48 (0.92-2.51) | 0.126 |
| Healthcare professional | 0.26 (0.17-0.40) | <0.001 |
| Unemployed | 0.59 (0.35-1.07) | 0.068 |
| Furloughed | 1.01 (0.54-2.05) | 0.979 |
| Unable to work | 1.58 (0.56-6.61) | 0.453 |
| Retired | 1.40 (0.90-2.19) | 0.136 |

[1] Adjusted for age and school or work situation
* School pupils excluded from analysis due to insufficient data.

For peer review only

**Table S7.** Adjusted incidence rate ratios for number of daily non-household contacts by select variables. Model intercept was 0.40 (95 %CI 0.34-0.47). Dispersion parameter of 1.07 (95%CI 1.00-1.13)

| | Multivariable analysis[1] | |
|---|---|---|
| | aIRR (95%CI) | p-value |
| Age | | |
| 0-9 | 0.69 (0.13-3.65) | 0.663 |
| 10-19 | 0.88 (0.49-1.64) | 0.688 |
| 20-29 | 1.14 (0.96-1.36) | 0.142 |
| 30-39 | 0.86 (0.76-0.97) | 0.012 |
| 40-49 | 0.90 (0.82-0.99) | 0.032 |
| 50-59 | 1.00 | - |
| 60-69 | 0.89 (0.79-1.00) | 0.047 |
| 70-79 | 0.95 (0.79-1.15) | 0.598 |
| 80+ | 1.24 (0.69-2.27) | 0.446 |
| Sex | | |
| Female | 1.00 | - |
| Male | 1.01 (0.93-1.11) | 0.762 |
| Prefer not to say | 0.53 (0.27-1.05) | 0.061 |
| Ethnicity | | |
| White | 1.00 | - |
| Mixed/Multiple ethnic groups | 1.08 (0.76-1.56) | 0.667 |
| Asian/Asian British | 0.53 (0.35-0.81) | 0.003 |
| Black/African/Carribean/Black British | 0.46 (0.17-1.23) | 0.135 |
| Other ethnic group | 0.53 (0.17-1.77) | 0.290 |
| Nation | | |
| England | 1.00 | - |
| Northern Ireland | 0.92 (0.59-1.47) | 0.708 |
| Scotland | 0.79 (0.67-0.94) | 0.006 |
| Wales | 1.23 (1.00-1.51) | 0.052 |
| Household size | | |
| 1 | 1.00 | - |
| 2 | 0.92 (0.83-1.02) | 0.133 |

| | Multivariable analysis[1] | |
|---|---|---|
| | aIRR (95%CI) | p-value |
| 3 | 1.00 (0.89-1.13) | 0.981 |
| 4 | 0.85 (0.75-0.96) | 0.010 |
| 5 | 0.98 (0.83-1.17) | 0.839 |
| 6+ | 0.88 (0.68-1.16) | 0.357 |
| Day of the week (contacts recorded) | | |
| Weekday | 1.00 | - |
| Weekend | 1.14 (0.99-1.31) | 0.074 |
| Left home yesterday | | |
| No | 1.00 | |
| Yes | 5.60 (4.94-6.36) | <0.001 |
| School or work situation | | |
| Employed - working from home | 1.00 | - |
| School pupil - studying at home | 0.84 (0.29-2.48) | 0.752 |
| School pupil - attending school | 2.96 (0.97-10.23) | 0.074 |
| College or University student | 0.86 (0.65-1.14) | 0.287 |
| Employed - going to place of work | 3.32 (3.02-3.66) | <0.001 |
| Self Employed | 1.63 (1.43-1.87) | <0.001 |
| Healthcare professional | 5.05 (4.25-6.03) | <0.001 |
| Unemployed | 1.11 (0.90-1.37) | 0.354 |
| Furloughed | 1.20 (0.98-1.48) | 0.074 |
| Unable to work | 0.96 (0.71-1.30) | 0.798 |
| Retired | 1.24 (1.09-1.42) | 0.001 |
| COVID-19 circumstance | | |
| Not self-isolating or shielding | 1.00 | - |
| Self-isolating - I have symptoms* | 4.07 (1.96-9.79) | 0.001 |
| Self-isolating - Someone in my household has symptoms | 1.30 (0.51-3.60) | 0.614 |
| Self-isolating - Someone in my support bubble has symptoms | 0.82 (0.21-3.34) | 0.780 |
| Self-isolating - precaution/told to do so by Test and Trace | 0.58 (0.43-0.79) | <0.001 |
| Shielding - I am a vulnerable individual | 0.81 (0.66-1.01) | 0.050 |



For peer review only - http://bmjopen.bmj.com/site/about/guidelines.xhtml

| | Multivariable analysis[1] | |
|---|---|---|
| | aIRR (95%CI) | p-value |
| Shielding - I live with a vulnerable individual | 0.79 (0.62-1.01) | 0.063 |
| Not sure | 0.55 (0.39-0.78) | 0.001 |

[1]Adjusted for age, sex, ethnicity, nation, household size, day of the week, left home, school or work situation and COVID-19 circumstance.

*This increased contact rate is due to one participant who was self-isolating with symptoms reporting a high number of contacts (*see results*).

For peer review only

**Table S8.** Association of participant characteristics with risk (adjusted odds ratios) of visiting another household (N = 4,030). Model intercept 0.11 (0.09-0.14).

| | Adjusted Odds Ratio (95%CI)[1] | p-value |
|---|---|---|
| Age | | |
| 10-19 | 0.75 (0.18-2.20) | 0.643 |
| 20-29 | 0.77 (0.47-1.21) | 0.277 |
| 30-39 | 0.85 (0.62-1.15) | 0.313 |
| 40-49 | 1.03 (0.82-1.30 | 0.805 |
| 50-59 | 1.00 | - |
| 60-69 | 1.09 (0.86-1.38) | 0.479 |
| 70-79 | 1.18 (0.79-1.72) | 0.413 |
| 80+ | 0.73 (0.11-2.73) | 0.682 |
| Sex | | |
| Female | 1.23 (0.98-1.56) | 0.073 |
| Male | 1.00 | - |
| Prefer not to say | 1.07 (0.17-3.92) | 0.926 |
| Part of a support bubble | | |
| No | 1.00 | - |
| Yes | 1.92 (1.61-2.28) | <0.001 |

[1]Adjusted for age, sex and whether a participants was a part of a support bubble.
*0-9 year olds excluded from analysis due to insufficient data.

For peer review only

**References**

1 Mossong J, Hens N, Jit M, *et al.* Social contacts and mixing patterns relevant to the spread of infectious diseases. *PLoS Med* 2008;**5**:e74.

2 Wasserman S, Faust K, Stanley (University of Illinois Wasserman, Urbana-Champaign). *Social Network Analysis: Methods and Applications*. Cambridge University Press 1994.

3 Leclerc QJ, Fuller NM, Knight LE, *et al.* What settings have been linked to SARS-CoV-2 transmission clusters? *Wellcome Open Res* 2020;**5**:83.

4 Jarvis CI, Van Zandvoort K, Gimma A, *et al.* Quantifying the impact of physical distance measures on the transmission of COVID-19 in the UK. *BMC Med* 2020;**18**:124.

5 Danon L, Read JM, House TA, *et al.* Social encounter networks: characterizing Great Britain. *Proc Biol Sci* 2013;**280**:20131037.

For peer review only

**Survey Questions - Round 1**

Q1
Are you aged 13 or over?

- ◯ Yes
- ◯ No

Q1.a
**Please make sure you agree to the following before continuing with the survey:**
You currently live in the UK;
You have read the Participant Information Sheet and fully understand what is expected of you within this study;
Your participation is voluntary and you are aware that you can stop the survey at any point; You understand that data submitted prior to closing the survey will be collected;
You consent to Lancaster University keeping the anonymised data for a period of 10-years after the study has finished;
If you are filling out the survey on behalf of someone else, please make sure you have their consent before continuing.

- ◯ **I consent to taking part in the CoCoNet study**

Q1.b
**If you are under the age of 13 we do ask that a parent or guardian fills out the survey with you.**

Please take the time to read through and discuss the *Information sheet for Children* together. The parent or guardian should also read through the more detailed *Participant Information Sheet*.

**Please make sure you both agree to the following before continuing with the survey:**
I live in the UK;
I have read and understood the information sheet(s);
I understand I can stop the survey at any point;
I understand that my answers will be kept for 10 years after the study has finished.

| | Child | Parent/Guardian |
|---|---|---|
| **I agree to take part in the CoCoNet study/ I consent to my child taking part in the study** | ☐ | ☐ |

For peer review only

Q2 Where in the UK do you currently live?

- England
- Northern Ireland
- Scotland
- Wales
- I do not live in the UK

For peer review only

Q3 What is your age?

- 0 - 9 years old
- 10 - 19 years old
- 20 - 29 years old
- 30 - 39 years old
- 40 - 49 years old
- 50 - 59 years old
- 60 - 69 years old
- 70 - 79 years old
- Aged 80 or over

Q4 What is your sex?

*The answer you give can be different from what is on your birth certificate.*

- Female
- Male
- Prefer not to say



For peer review only - http://bmjopen.bmj.com/site/about/guidelines.xhtml

Q5 Which of the following best describes your ethnicity?

- ◯ English / Welsh / Scottish / Northern Irish / British
- ◯ Irish
- ◯ Gypsy or Irish Traveller
- ◯ Any other White background
- ◯ White and Black Caribbean
- ◯ White and Black African
- ◯ White and Asian
- ◯ Any other Mixed / Multiple ethnic background
- ◯ Indian
- ◯ Pakistani
- ◯ Bangladeshi
- ◯ Chinese
- ◯ Any other Asian background
- ◯ African
- ◯ Caribbean
- ◯ Any other Black / African / Caribbean background
- ◯ Arab
- ◯ Any other ethnic group
- ◯ Prefer not to say

For peer review only

Q6 What is the first part of your home postcode?
*For example, if your home postcode was LA1 4YW then you would enter LA1.*

______________________________________________________________

Q7 Which type of accommodation best describes your home?

- ◯ Flat, maisonette or apartment
- ◯ House or bungalow
- ◯ Mobile or temporary structure
- ◯ Assisted living facility
- ◯ Care home
- ◯ Other

For peer review only

Q8
What is your current school or work situation?

- ◯ School pupil - studying at home
- ◯ School pupil - still attending school
- ◯ College or University student
- ◯ Employed - working from home
- ◯ Employed - still going to place of work
- ◯ Self Employed
- ◯ Healthcare professional
- ◯ Unemployed
- ◯ Furloughed
- ◯ Unable to work
- ◯ Retired
- ◯ Other

For peer review only

Q9 Currently, do you regularly meet members of the general public as part of your job?

- ◯ Yes
- ◯ No



For peer review only - http://bmjopen.bmj.com/site/about/guidelines.xhtml

Q10
Are you self-isolating or shielding because of COVID-19?
*A vulnerable individual here refers to a clinically extremely vulnerable person.*

- ◯ I am not self-isolating or shielding
- ◯ Self Isolating - I have symptoms of COVID
- ◯ Self Isolating - Someone in my household has symptoms of COVID
- ◯ Self Isolating - Someone in my support bubble has symptoms of COVID
- ◯ Self Isolating - As a precaution / told to do so by Test and Trace
- ◯ Shielding - I am a vulnerable individual
- ◯ Shielding - I live with a vulnerable individual
- ◯ Not sure

Q11
How many other people currently live with you at home?

- ◯ 0 - I live alone
- ◯ 1
- ◯ 2
- ◯ 3
- ◯ 4
- ◯ 5 or more

For peer review only

Q12
How many people of each age group live with you at home?
*Do not include yourself.*

*Drop down options of 0, 1, 2, 3, 4, 5 or more for each age group.*

0 - 9 year olds
10 - 19 year olds
20 - 29 year olds
30 - 39 year olds
40 - 49 year olds
50 - 59 year olds
60 -69 year olds
70 - 79 year olds
Aged 80 or over

Q13
Have you formed a support bubble with another household?
*A single-person household can join with one other household and interact without maintaining social distance.*

◯ Yes

◯ No

Q14
How many people of each age group are part of your support bubble?
*Do not include your own household members.*

*Drop down options of 0, 1, 2, 3, 4, 5 or more for each age group.*

0 - 9 year olds
10 - 19 year olds
20 - 29 year olds
30 - 39 year olds
40 - 49 year olds
50 - 59 year olds
60 -69 year olds
70 - 79 year olds
Aged 80 or over

For peer review only

Q15
Thinking about the past 7 days, on how many of these days did you meet someone from your support bubble?

- ◯ None
- ◯ 1 day
- ◯ 2 days
- ◯ 3 days
- ◯ 4 days
- ◯ 5 days
- ◯ 6 days
- ◯ 7 days
- ◯ Not sure

For peer review only

Q16

Did you leave your home or property yesterday?

*Do not include going into your private garden, but do include visits to shared or communal gardens or spaces.*

- ◯ Yes
- ◯ No

Q17

Where did you go yesterday? Tick all that apply.

- ☐ Visited the home of someone else
- ☐ My school or workplace
- ☐ Doctor's surgery or healthcare facility
- ☐ Supermarket or convenience store
- ☐ Other shops or retail spaces (e.g. garden centre, clothing shops, drive-through food outlets)
- ☐ Restaurant, café or pub
- ☐ For a walk or exercise
- ☐ Other - *please do not include any identifying information*

________________________________________

For peer review only

Q18
What modes of transport did you use yesterday? Tick all that apply.

- ☐ I walked or cycled
- ☐ I travelled in a car by myself
- ☐ I travelled in a car with another person(s)
- ☐ I took a bus, tram or train
- ☐ I took an aeroplane or ferry

For peer review only

Q19
Not including those that you live with, how many people did you meet yesterday?
*Only include those you had a face-to-face conversation with.*

- ○ None
- ○ 1
- ○ 2
- ○ 3
- ○ 4
- ○ 5
- ○ 6
- ○ 7
- ○ 8
- ○ 9
- ○ 10
- ○ 11
- ○ 12
- ○ 13
- ○ 14
- ○ 15 or more

For peer review only

Q20
Please tell us about each of the people you met yesterday.
*Information collected for up to 14 contacts.*

| How old were they? *Please estimate the person's age if you are unsure.* | | | | | | | | Did you meet this person indoors? *For example, in a shop, office or house.* | | Did anyone that you live with also meet this person yesterday? | | |
|---|---|---|---|---|---|---|---|---|---|---|---|---|
| 0 - 4 | 5 - 9 | 10 - 19 | 20 - 39 | 40 - 59 | 60 - 69 | 70 - 79 | 80 + | Yes | No | Yes | No | Not sure |

Q21
Please tell us about each of the people you met yesterday.
*Information collected for up to 14 contacts.*

| How old were they? *Please estimate the person's age if you are unsure.* | | | | | | | | Did you meet this person indoors? *For example, in a shop, office or house.* | |
|---|---|---|---|---|---|---|---|---|---|
| 0 - 4 | 5 - 9 | 10 - 19 | 20 - 39 | 40 - 59 | 60 - 69 | 70 - 79 | 80 + | Yes | No |

For peer review only

Q22
How many people of each age group did you meet yesterday?
*Drop down choice of integers 1 - 19 or '20 or more' for each age group.*

0-4 year olds

5-9 year olds

10-19 year olds

20-39 year olds

40-59 year olds

60-69 year olds

70-79 year olds

Aged 80 or over

Q23
Did you meet these people indoors or outdoors?
*For example, meeting someone indoors could be in a shop, office or house etc.*

- ◯ I met everyone indoors
- ◯ I met most people indoors
- ◯ I met most people outdoors
- ◯ I met everyone outdoors
- ◯ Not sure

For peer review only

Q24
Were you able to maintain social distance from everyone you met yesterday?
*Do not include people that you live with or those in your support bubble. Please refer to the government advice for the recommended social distance in your area.*

- ○ Yes, all of the time
- ○ More than half of the time
- ○ Less than half of the time
- ○ No, none of the time
- ○ Not sure

Q25 Of the people you live with, how many people stayed at home all day yesterday?

- ○ None
- ○ 1
- ○ 2
- ○ 3
- ○ 4
- ○ 5 or more
- ○ Not sure

For peer review only

Q26 Thinking about the past 7 days, on how many of these days did you leave your home or property?

- None
- 1 day
- 2 days
- 3 days
- 4 days
- 5 days
- 6 days
- 7 days
- Not sure

Q27 What was the furthest distance from home you travelled over the past 7 days?

- Less than 2 miles (3 km)
- 2 - 9 miles (3 - 15 km)
- 10 - 19 miles (16 - 31 km)
- 20 - 49 miles (32 - 79 km)
- 50 miles (80 km) or more

For peer review only

STROBE Statement—Checklist of items that should be included in reports of ***cross-sectional studies***

| | Item No | Recommendation | Page No |
|---|---|---|---|
| **Title and abstract** | 1 | (*a*) Indicate the study's design with a commonly used term in the title or the abstract | 1 |
| | | (*b*) Provide in the abstract an informative and balanced summary of what was done and what was found | 2 |
| **Introduction** | | | |
| Background/rationale | 2 | Explain the scientific background and rationale for the investigation being reported | 4 |
| Objectives | 3 | State specific objectives, including any prespecified hypotheses | 5 |
| **Methods** | | | |
| Study design | 4 | Present key elements of study design early in the paper | 4/5 |
| Setting | 5 | Describe the setting, locations, and relevant dates, including periods of recruitment, exposure, follow-up, and data collection | 4/5 |
| Participants | 6 | (*a*) Give the eligibility criteria, and the sources and methods of selection of participants | 4/5 |
| Variables | 7 | Clearly define all outcomes, exposures, predictors, potential confounders, and effect modifiers. Give diagnostic criteria, if applicable | 5 |
| Data sources/ measurement | 8* | For each variable of interest, give sources of data and details of methods of assessment (measurement). Describe comparability of assessment methods if there is more than one group | 5/6 |
| Bias | 9 | Describe any efforts to address potential sources of bias | 5 |
| Study size | 10 | Explain how the study size was arrived at | 4 |
| Quantitative variables | 11 | Explain how quantitative variables were handled in the analyses. If applicable, describe which groupings were chosen and why | 5/6 |
| Statistical methods | 12 | (*a*) Describe all statistical methods, including those used to control for confounding | 5/6 |
| | | (*b*) Describe any methods used to examine subgroups and interactions | 6 |
| | | (*c*) Explain how missing data were addressed | 6 |
| | | (*d*) If applicable, describe analytical methods taking account of sampling strategy | NA |
| | | (*e*) Describe any sensitivity analyses | NA |
| **Results** | | | |
| Participants | 13* | (a) Report numbers of individuals at each stage of study—eg numbers potentially eligible, examined for eligibility, confirmed eligible, included in the study, completing follow-up, and analysed | 6/7/8 /10/11 |
| | | (b) Give reasons for non-participation at each stage | 7/8/10/11 |
| | | (c) Consider use of a flow diagram | NA |
| Descriptive data | 14* | (a) Give characteristics of study participants (eg demographic, clinical, social) and information on exposures and potential confounders | 6 |
| | | (b) Indicate number of participants with missing data for each variable of interest | 7/8/10/11 |
| Outcome data | 15* | Report numbers of outcome events or summary measures | 7/8/9/10/11 |

For peer review only

| | | | |
|---|---|---|---|
| Main results | 16 | (*a*) Give unadjusted estimates and, if applicable, confounder-adjusted estimates and their precision (eg, 95% confidence interval). Make clear which confounders were adjusted for and why they were included | 15/16 |
| | | (*b*) Report category boundaries when continuous variables were categorized | NA |
| | | (*c*) If relevant, consider translating estimates of relative risk into absolute risk for a meaningful time period | NA |
| Other analyses | 17 | Report other analyses done—eg analyses of subgroups and interactions, and sensitivity analyses | 12/13 |
| **Discussion** | | | |
| Key results | 18 | Summarise key results with reference to study objectives | 14/15 |
| Limitations | 19 | Discuss limitations of the study, taking into account sources of potential bias or imprecision. Discuss both direction and magnitude of any potential bias | 15 |
| Interpretation | 20 | Give a cautious overall interpretation of results considering objectives, limitations, multiplicity of analyses, results from similar studies, and other relevant evidence | 15 |
| Generalisability | 21 | Discuss the generalisability (external validity) of the study results | 15 |
| **Other information** | | | |
| Funding | 22 | Give the source of funding and the role of the funders for the present study and, if applicable, for the original study on which the present article is based | 16 |

*Give information separately for exposed and unexposed groups.

**Note:** An Explanation and Elaboration article discusses each checklist item and gives methodological background and published examples of transparent reporting. The STROBE checklist is best used in conjunction with this article (freely available on the Web sites of PLoS Medicine at http://www.plosmedicine.org/, Annals of Internal Medicine at http://www.annals.org/, and Epidemiology at http://www.epidem.com/). Information on the STROBE Initiative is available at www.strobe-statement.org.

For peer review only

BMJ Open

# Social mixing patterns in the UK following the relaxation of COVID-19 pandemic restrictions, July to August 2020: a cross-sectional online survey.



| | |
|---|---|
| Journal: | *BMJ Open* |
| Manuscript ID | bmjopen-2021-059231.R1 |
| Article Type: | Original research |
| Date Submitted by the Author: | 21-Jul-2022 |
| Complete List of Authors: | Bridgen, Jessica; Lancaster University, Lancaster Medical School<br>Jewell, Christopher ; Lancaster University Faculty of Health and Medicine, Lancaster Medical School<br>Read, Jonathan; Lancaster University Faculty of Health and Medicine, Lancaster Medical School |
| <b>Primary Subject Heading</b>: | Epidemiology |
| Secondary Subject Heading: | Infectious diseases, Public health |
| Keywords: | COVID-19, EPIDEMIOLOGY, Epidemiology < INFECTIOUS DISEASES, Public health < INFECTIOUS DISEASES |
| | |



SCHOLARONE™ Manuscripts

*I, the Submitting Author has the right to grant and does grant on behalf of all authors of the Work (as defined in the below author licence), an exclusive licence and/or a non-exclusive licence for contributions from authors who are: i) UK Crown employees; ii) where BMJ has agreed a CC-BY licence shall apply, and/or iii) in accordance with the terms applicable for US Federal Government officers or employees acting as part of their official duties; on a worldwide, perpetual, irrevocable, royalty-free basis to BMJ Publishing Group Ltd ("BMJ") its licensees and where the relevant Journal is co-owned by BMJ to the co-owners of the Journal, to publish the Work in this journal and any other BMJ products and to exploit all rights, as set out in our licence.*

*The Submitting Author accepts and understands that any supply made under these terms is made by BMJ to the Submitting Author unless you are acting as an employee on behalf of your employer or a postgraduate student of an affiliated institution which is paying any applicable article publishing charge ("APC") for Open Access articles. Where the Submitting Author wishes to make the Work available on an Open Access basis (and intends to pay the relevant APC), the terms of reuse of such Open Access shall be governed by a Creative Commons licence – details of these licences and which Creative Commons licence will apply to this Work are set out in our licence referred to above.*

*Other than as permitted in any relevant BMJ Author's Self Archiving Policies, I confirm this Work has not been accepted for publication elsewhere, is not being considered for publication elsewhere and does not duplicate material already published. I confirm all authors consent to publication of this Work and authorise the granting of this licence.*

For peer review only

# Social mixing patterns in the UK following the relaxation of COVID-19 pandemic restrictions, July to August 2020: a cross-sectional online survey

Jessica RE Bridgen; Lancaster Medical School, Lancaster University, Lancaster, UK, LA1 4YW
Chris P Jewell; Lancaster Medical School, Lancaster University, Lancaster, UK, LA1 4YW
Jonathan M Read; Lancaster Medical School, Lancaster University, UK, LA1 4YW
jonathan.read@lancaster.ac.uk (corresponding author)

Word Count: 3350

For peer review only

**Abstract**

**Objectives:** To quantify and characterize non-household contact and to identify the effect of shielding and isolating on contact patterns.

**Design:** Cross-sectional study.

**Setting and participants:** Anyone living in the UK was eligible to take part in the study. We recorded 5,143 responses to the online questionnaire between 28 July and 14 August 2020.

**Outcome measures:** Our primary outcome was the daily non-household contact rate of participants. Secondary outcomes were propensity to leave home over a 7 day period, whether contacts had occurred indoors or outdoors locations visited, furthest distance travelled from home, ability to socially distance, and membership of support bubble.

**Results:** The mean rate of non-household contacts per person was 2.9 $d^{-1}$. Participants attending a workplace (adjusted incidence rate ratio (aIRR) 3.33, 95%CI 3.02 to 3.66), self-employed (aIRR 1.63, 95%CI 1.43 to 1.87) or working in healthcare (aIRR 5.10, 95%CI 4.29 to 6.10) reported significantly higher non-household contact rates than those working from home. Participants self-isolating as a precaution or following Test and Trace instructions had a lower non-household contact rate than those not self-isolating (aIRR 0.58, 95%CI 0.43 to 0.79). We found limited evidence that those shielding had reduced non-household contacts compared to non-shielders.

**Conclusion:** The daily rate of non-household interactions remained lower than pre-pandemic levels measured by other studies, suggesting continued adherence to social distancing guidelines. Individuals attending a workplace in-person or employed as healthcare professionals were less likely to maintain social distance and had a higher non-household contact rate, possibly increasing their infection risk. Shielding and self-isolating individuals required greater support to enable them to follow the government guidelines and reduce non-household contact and therefore their risk of infection.

For peer review only

**Strengths and Limitations**

- Large-scale cross-sectional study.
- This study provides detailed information on non-household contact and associated behaviours.
- The study period corresponds with the start of epidemic growth, behaviours measured could provide insight into the level of social mixing needed to support epidemic growth.
- As there was no active recruitment process certain demographic groups are underrepresented and the study may suffer from recruitment bias.
- Social contacts were self-reported by participants and were therefore subject to recall bias.

For peer review only



For peer review only - http://bmjopen.bmj.com/site/about/guidelines.xhtml

## INTRODUCTION

On 31 January 2020, the first two cases of COVID-19 were recorded in the United Kingdom (UK), followed by a rapid rise in identified cases and hospitalised patients. On 23 March 2020, a range of social distancing measures were implemented across the UK (lockdown), aiming to reduce interpersonal contact between households and reduce transmission of SARS-CoV-2. Schools were closed to pupils, with the exception of children of key workers. People were only allowed to leave their homes to shop for basic necessities, to exercise once a day, for medical reasons, and to travel to work if working from home was not possible.[1] By July 2020, many businesses, including shops, restaurants and pubs, had reopened. Support bubbles had been introduced, allowing for a single-adult household to interact with another household of any size.[2] International travel was permitted, following the introduction of travel corridors on 10 July 2020, which enabled passengers to travel to England from certain countries without self-isolating.[3] The UK government's 'Eat Out to Help Out' scheme, which ran from 3 to 31 August 2020, encouraged people to dine out.[4] Some social distancing restrictions remained in place, including maintaining a 2 metre distance between individuals (excluding household members or members of a support bubble), the wearing of face coverings on public transport and in shops, and limits on how many people could meet indoors and outdoors. [5–8] Whilst some people in the UK began to return to work, schools remained closed. A marked decrease in case incidence was seen during April 2020, and cases remained low until the onset of the second wave in August 2020.

Epidemics are largely driven by social mixing patterns and their quantification is useful for transmission modelling purposes, as well as assessing adherence to regulations and identifying sociodemographic factors associated with heterogeneities in contact rate.[9–11] The apparent association between social distancing restrictions and reduced case incidence indicates that a nuanced understanding of how individuals' contact patterns vary could inform behavioural interventions for the remainder of the outbreak. Previous contact studies have provided estimates for age-specific contact rates in Great Britain and the UK.[11–13] A cross-sectional survey of UK adults early on during the lockdown beginning in March found a substantial reduction in daily contact between people.[14]

We conducted a cross-sectional online survey between 28 July and 14 August 2020 to measure the mobility of people living in the UK, which locations people were frequenting, and the number of non-household contacts people were making. We aimed to quantify non-household contact behaviour and adherence to self-isolation and shielding guidance. The study period coincided with the start of the second wave of SARS-CoV-2 infection in the UK, when hospital admissions for COVID-19 were at their lowest rate since April.[15]

## METHODS

### Survey Methodology

Data collection was conducted through an anonymous online questionnaire; the study was branded the CoCoNet (COVID-19 Contact Network) survey. The survey was open to anyone living in the UK at the time of the survey. There was no lower age limit for participation, with children under 13 required to complete the survey with a parent or guardian. The inclusion

For peer review only

criteria for participants were that they completed the question on residency location and that they were resident in the UK at the time of the survey.
The survey was promoted through a university press release, engagement with the media, and posts on social media directing potential participants to the study website: https://www.lancaster.ac.uk/health-and-medicine/research/coconet-study/.

Demographic information from participants, including age, sex, ethnicity, home location (first part of postcode) and their employment or school situation, was collected. Participants were asked about their household size, as well as the formation and size of support bubbles they may belong to. Participants were asked about their activities on the previous day (the contact reporting day), including whether they left their household and the number and characteristics of non-household contacts encountered. The questionnaire is presented in Supplementary Material and the dataset is publicly available[16]

To reduce participant burden, a triage question on how many people participants had met the previous day determined the level of information collected on contacts. Participants reporting fewer than 15 contacts were asked to estimate the age of each contact they made, whether they met the contact indoors or outdoors, and if anyone from their household had also met that contact the same day. Participants who reported 15 or more contacts were asked to estimate the number of contacts made with different age groups, and whether they had met most of their contacts indoors or outdoors.

Responses recorded between 00:00BST 28 July and 18:00BST 14 August 2020 were included in the analysis. Partial responses to the survey were analysed if the first compulsory question asking which part of the UK a participant resided in was answered. If a participant exited the online survey early, we used their responses up to and including the last question they saw.

**Primary and secondary outcome measurements**

Our primary outcome was non-household contact rate. A non-household contact was defined as someone with whom the participant had a face-to-face conversation with, excluding members of their own household. A participant who remained at home could still make non-household contacts by having visitors to their home.

Secondary outcomes were whether contacts occurred indoors or outdoors, propensity to leave home over a 7 day period, ability to socially distance, locations visited, furthest distance travelled from home, and membership of support bubble.

**Descriptive analysis**

Representativeness was assessed by visual comparison of participant demographics with respective Office for National Statistics (ONS) 2019 mid-year estimates.[17,18] The mean number of non-household contacts was calculated and stratified by age, sex and household size, and was compared to reported values from other social contact surveys. Adherence to social distancing guidance was assessed by calculating the proportion of participants who left home in the past 7 days, the distribution of furthest distance travelled in the past 7 days, and the proportion of participants who felt able to maintain a recommended physical distance during contact with others. Non-responses were excluded from analyses.


For peer review only - http://bmjopen.bmj.com/site/about/guidelines.xhtml

## Predictors of contact frequency

To identify characteristics of the participant associated with their rate of daily non-household contact we fitted a negative binomial model to the daily number of non-household contacts reported by participants. Explanatory variables included in the model a priori were: age; sex; ethnicity; nation of residence (England, Northern Ireland, Scotland or Wales); household size; dwelling type; whether the contact reporting day was a weekend or week day; whether the participant had left their home on the contact reporting day; participant's working situation; participant's COVID-19 circumstance. To support our hypothesis-driven choice of model parameters, we also conducted a forward stepwise model selection process, with our previously selected explanatory variables used as candidate variables; see supplementary materials. Statistical analyses were conducted using R version 4.0.2.[19]

## Patient and public involvement statement

Patients or the public were not involved in the design, or conduct, or reporting, or dissemination plans of our research. However, as the online survey was promoted via social media, members of the public were free to further promote it via social media links.

# RESULTS

## Participant demographics

We received 5,383 survey responses recorded between 28 July 2020 and 14 August 2020; 5,143 responses met our inclusion criteria.[16] Most participants were aged 40-59 (55.3%, 2813/5090); Table 1, Figure 1A. We recorded fewer responses from participants in the youngest age groups, 0-9 year olds (0.1%, 5/5090) and 10-19 year olds (0.7%, 38/5090), and in the oldest age group, aged 80+ (0.4%, 21/5090). Males, non-white ethnicities, and residents of Northern Ireland and Wales were under-represented in our sample.

For peer review only

**Table 1:** Participant demography and UK ONS 2019 mid-year estimates. N is the number of participants who provided a response to the question.

| | **Number of participants (%)** | **UK ONS mid-year estimates (2019)[*]** |
|---|---|---|
| Age group (N = 5,090)[†] | | |
| 0-9 | 5 (0.1%) | 12.0% |
| 10-19 | 38 (0.7%) | 11.4% |
| 20-29 | 256 (5.0%) | 13.0% |
| 30-39 | 598 (11.7%) | 13.3% |
| 40-49 | 1183 (23.2%) | 12.6% |
| 50-59 | 1630 (32.0%) | 13.6% |
| 60-69 | 1065 (20.9%) | 10.7% |
| 70-79 | 294 (5.8%) | 8.4% |
| 80+ | 21 (0.4%) | 5.0% |
| Sex (N = 5,090)[†] | | |
| Female | 4017 (78.9%) | 50.6% |
| Male | 1051 (20.6%) | 49.4% |
| Prefer not to say | 22 (0.4%) | - |
| Ethnicity (N = 5,090) | | |
| White | 4880 (95.9%) | 86.0% |
| Mixed/Multiple ethnic groups | 49 (1.0%) | 2.2% |
| Asian/Asian British | 50 (1.0%) | 7.5% |
| Black/African/Caribbean/Black British | 11 (0.2%) | 3.3% |
| Other ethnic groups | 7(0.1%) | 1.0% |
| Prefer not to say | 16 (0.3%) | - |
| No response | 77 (1.5%) | - |
| Nation (N = 5,143)[†] | | |
| England | 4714 (91.7%) | 84.3% |
| Northern Ireland | 33 (0.6%) | 2.8% |
| Scotland | 254 (4.9%) | 4.7% |
| Wales | 142 (2.8%) | 8.2% |
| Household size (N = 5,073)[†] | | |
| 1 | 878 (17.3%) | 29.5% |
| 2 | 1911 (37.7%) | 34.5% |
| 3 | 987 (19.5%) | 15.4% |
| 4 | 907 (17.9%) | 13.9% |
| 5 | 287 (5.7%) | 4.5% |
| 6+ | 103 (2.0%) | 2.1% |

For peer review only

175 * Ethnicity estimates from 2011 census data.
176 † Question required a response from participants to progress through the online survey.

For peer review only

**Mobility**

We found 33.7% (95% confidence interval (CI) 32.4 to 35.0) of participants left their home every day over a 7-day period; Table 2. Over the same time period, most participants travelled less than 10 miles from home, but some longer-range travel (50+ miles) occurred.

For peer review only

**Table 2:** Ability of participants to social distance, membership and size of support bubbles, locations visited and mobility of participants. N is the number of participants who provided a response to the question.

| | **Number of participants (%)** |
|---|---|
| Maintaining social distance yesterday (N = 3,249)† | |
| All of the time | 1910 (58.8%) |
| More than half of the time | 934 (28.7%) |
| Less than half of the time | 296 (9.1%) |
| None of the time | 89 (2.7%) |
| Not sure | 20 (0.6%) |
| Part of a support bubble (N = 5,066)† | |
| Yes | 2029 (40.1%) |
| No | 3037 (59.9%) |
| Support bubble size (N = 2,011) | |
| 1 | 866 (43.1%) |
| 2 | 560 (27.8%) |
| 3 | 229 (11.4%) |
| 4 | 201 (10.0%) |
| 5+ | 155 (7.7%) |
| No response | 18 |
| Frequency of leaving home in past 7 days (N = 4,896) | |
| 0 days | 82 (1.7%) |
| 1 day | 281 (5.7%) |
| 2 days | 518 (10.6%) |
| 3 days | 605 (12.4%) |
| 4 days | 568 (11.6%) |
| 5 days | 650 (13.3%) |
| 6 days | 537 (11.0%) |
| 7 days | 1650 (33.7%) |
| Not sure | 5 (0.1%) |
| No response | 30 |
| Locations visited yesterday (N = 4,034) | |
| Someone's home | 615 (15.2%) |
| School or workplace | 612 (15.2%) |
| Doctor's surgery or healthcare facility | 182 (4.5%) |
| Supermarket or convenience store | 1473 (36.5%) |
| Other shops or retail spaces | 596 (14.8%) |

For peer review only



For peer review only - http://bmjopen.bmj.com/site/about/guidelines.xhtml

| | Number of participants (%) |
|---|---|
| Restaurant, café or pub | 553 (13.7%) |
| For a walk or exercise | 2178 (54.0%) |
| Other | 808 (20.0%) |
| No response | 0 |
| Furthest distance travelled in past 7 days (N = 4,913) | |
| Under 2 miles | 886 (18.0%) |
| 2 - 9 miles | 1682 (34.2%) |
| 10 - 19 miles | 848 (17.3%) |
| 20 - 49 miles | 669 (13.6%) |
| 50+ miles | 828 (16.9%) |
| No response | 13 |

184 † Question required a response from participants to progress through the online survey.

For peer review only

### Non-household contacts

A total of 14,388 non-household contacts were recorded by 5,037 participants. The mean rate of non-household contacts was 2.9 $d^{-1}$ (95%CI 2.7 to 3.0). This is a notably lower rate of non-household contact than recorded from pre-pandemic surveys; Supplementary Table 1. We found 33.4% (95%CI 32.1 to 34.7) of participants made no non-household contacts. The degree distribution of non-household contacts has a long right-hand tail (95th percentile: 10 contacts $d^{-1}$, maximum 130 contacts $d^{-1}$); Figure 1B. We also quantified the non-household contact rate of household members of participants; see supplementary materials.

Mean non-household contact rate varied by age and was highest among 10-19 year olds (mean 3.6, 95%CI 1.6 to 6.5); Figure 1C. We found moderate assortative mixing by age, in line with both current and pre-pandemic contact studies; Supplementary Figure 1A. We found that the mean daily non-household contact rate by participant age group was substantially lower when compared to pre-pandemic POLYMOD study; see supplementary materials, Supplementary Figure 1B. A notable decrease in contact rate was found between people aged under 60 mixing with others aged under 60, with the largest reduction in contact rate seen across all age groups when mixing with 0-19 year olds; Supplementary Figure 1B.

### Participant characteristics and non-household contact rate

We identified the association of participant characteristics with the rate of non-household contact using a multiple regression model; Figure 2, Supplementary Table 2. The candidate variable dwelling type was not selected by the model selection process; Supplementary Table 3. We found no association of non-household contact rate with sex or day of the week. Contact rate varied by participant age: participants aged 30-39 (adjusted incidence rate ratio (aIRR) 0.86, 95% CI 0.76 to 0.97), aged 40-49 (aIRR 0.90, 95%CI 0.82 to 1.00) and those aged 60-69 (aIRR 0.89, 95%CI 0.79 to 1.00) reported a lower rate of contact than participants aged 50-59. We found that Asian and Asian British participants had a lower rate of contact than White participants (aIRR 0.54, 95%CI 0.36 to 0.82). Participants residing in Scotland had a lower contact rate than those living in England (aIRR 0.80, 95%CI 0.68 to 0.95), whereas participants in Wales had a higher contact rate (aIRR 1.22, 95%CI 0.99 to 1.50).

Leaving home was associated with a higher non-household contact rate than staying at home (aIRR 5.58, 95%CI to 4.92 to 6.33). Attending a workplace (aIRR 3.33, 95%CI 3.02 to 3.66), being self-employed (aIRR 1.63, 95%CI 1.43 to 1.87) or working in healthcare (aIRR 5.10, 95%CI 4.29 to 6.10) was associated with a significantly higher rate of non-household contact than working at home.

### Social distancing characteristics of shielding and self-isolating individuals

There were 353 (6.9%, 353/5073) participants who reported their COVID circumstance to be shielding, either due to being a vulnerable individual or living with a vulnerable individual. Additionally, 136 (2.7%, 136/5073) participants reported their COVID circumstance as self-isolating. Shielding individuals tended to be older than non-shielding individuals; Supplementary Table 4.

Shielding and self-isolating participants were less likely to leave their home compared to those reporting their situation to be 'not self-isolating or shielding': 58.6% (95%CI 53.2 to 63.8) of shielding individuals, 52.6% (95%CI 43.8 to 61.2) of self-isolating individuals, and

For peer review only

82.7% (95%CI 81.6 to 83.8) of other participants reported leaving their home during the contact day; Supplementary Table 4. The majority of shielding and self-isolating participants adhered to contemporary social distancing guidelines: 70.1% (95%CI 62.5 to 76.9) of shielding participants and 73.6% (95%CI 59.7 to 84.7) of self-isolating participants reported maintaining social distance at all time with contacts met the previous day; Supplementary Table 4.

Shielding and self-isolating individuals made fewer contacts per day outside of the household than non-shielding or isolating individuals. The unadjusted rate of non-household contact was 1.3 $d^{-1}$ (95%CI 1.1 to 1.5) amongst shielding participants, 1.2 $d^{-1}$ (95%CI 0.7 to 2.1) for self-isolating participants and 3.1 $d^{-1}$ (95%CI 2.9 to 3.2) for participants who were not self-isolating or shielding. After adjusting for other variables, we found vulnerable individuals shielding had a marginally lower non-household contact rate than those not shielding or self-isolating (aIRR 0.82, 95%CI 0.66 to 1.01). Those self-isolating as a precaution or under Test and Trace instructions had a lower non-household contact rate than individuals not shielding or self-isolating (aIRR 0.58, 95%CI 0.43 to 0.79); Figure 2. Individuals who reported as self-isolating with symptoms had a higher rate of non-household contact than those not self-isolating or shielding (aIRR 4.05, 95%CI 1.94 to 9.72). However, a single participant in this group reported a very large number of contacts on their contact day. This is not necessarily an example of non-adherence to social distancing guidance, as contact day and current day are different days. Our questionnaire design asked about contact on the day prior to completing the survey, which would be the day of their current COVID-19 situation. When we exclude this individual from our analysis we found no significant difference in contact rate; see Supplementary Table 5.

**Ability to maintain social distancing**

Participants were asked how much of the time they were able to maintain social distance from everyone they had met the previous day, excluding members of their household and support bubble. We found 58.8% (95%CI 57.1 to 60.5) of participants felt able to maintain social distancing at all times, while 2.7% (95%CI 2.2 to 3.4) felt unable to maintain social distance at any time. We found that age and employment situation were associated with being able to 'maintain social distance more than half of the time'; Supplementary Table 6. Participants aged 30-39 felt less able to maintain social distance more than half of the time compared to 50-59 year olds (adjusted odds ratio (aOR) 0.66, 95%CI 0.46 to 0.95). Healthcare professionals (aOR 0.26, 95%CI 0.17 to 0.40) and those attending their workplace in-person (aOR 0.71, 95%CI 0.53 to 0.96) were less likely to be able to maintain social distance than those working from home.

**Location of encounters**

Transmission risk of SARS-CoV-2 is thought to be greater in enclosed, non-ventilated spaces and lower in outdoor environments.[20] To assess how interactions may be distributed by these settings, we asked participants reporting fewer than 15 individual contacts whether each contact was made indoors or outdoors, and asked all participants if they met all or the majority of contacts indoors or outdoors. The distribution of contacts by indoor/outdoor setting was bimodal: nearly half of participants reported meeting all of their non-household contacts indoors (48.8%, 95%CI 47.0 to 50.6), while 33.7% (95%CI 32.1 to 35.4) of participants reported meeting all of their non-household contacts outdoors. We also explored the non-household contacts of participants that remained at home (visitors) and the characteristics



For peer review only - http://bmjopen.bmj.com/site/about/guidelines.xhtml

associated with visiting another household; see supplementary materials, Supplementary Table 7.

## DISCUSSION

We found the daily rate of social contact was considerably lower than that measured prior to 2020 in similar but non-identical studies, despite our study period corresponding to a time when the COVID-19 pandemic social distancing restrictions were at their most relaxed during 2020 in the UK.[11–13,21] The Comix study of UK social contact rates reported a greatly reduced rate in March 2020 which increased during summer 2020, with the highest rate of contact recorded in August remaining markedly lower than pre-pandemic contact rate estimates.[22] Social contact studies outside of the UK also reported low daily contact rates in 2020. [23–25] A similar increase in contact rate following lockdown was observed by Latsuzbaia *et al.* in Luxembourg.[26]

Contact rates and ability to follow social distancing guidelines was associated with age and occupation. The older age groups (70-79, 80+), those at highest risk of severe COVID-19 outcomes, had the lowest non-household contact rates, and they mixed most often with 20-59 year olds. Individuals attending a workplace, or those self-employed or working in healthcare, had a higher daily non-household contact rate than those working from home, representing additional potential infection risk. A small proportion of participants reported making a large number (more than 50) of non-household contacts; these were exclusively participants who reported their employment situation as either attending their workplace in-person or working as a healthcare professional. Although the UK government was encouraging people to return to work at this time, we found that a high proportion of employed individuals (70.0%, excluding healthcare workers and those self-employed) continued to work from home.[27] In contrast to pre-pandemic contacts surveys, we found no association between non-household contact rate and day of the week.[11,13]

Black and Asian individuals have been shown to be at increased risk of SARS-CoV-2 infection in comparison to White individuals, possibly due to larger households, being more likely to be employed as essential workers, and less able to work from home.[28,29] However, after accounting for home-working, we found that individuals of Asian and Asian British ethnicity had a significantly lower non-household contact rate than White participants. This suggests that workplaces may be more dominant as a source of infection for these individuals than previously thought. [30]

The majority of participants reported being able to maintain social distance from others more than half of the time and very few participants reported failing to maintaining social distance at all, a similar observation made in a UK behavioural cohort.[31] Healthcare professionals and employees attending their workplace in-person were less able to maintain physical distance from people they encountered than people working from home. This highlights the increased risk of infection that some workers may face; occupations which require employees to interact closely with a large number of people are associated with an increased likelihood of exposure to COVID-19 and clusters of cases developing at a workplace.[32–34]


For peer review only - http://bmjopen.bmj.com/site/about/guidelines.xhtml

We found some evidence of non-adherence to self-isolating and shielding guidelines, with a high proportion of self-isolating and shielding participants leaving their home the previous day. Smith *et al*. also found low adherence to isolation instructions among the UK population during March through August 2020.[35] We found that a large proportion of self-isolating and shielding participants (including those living with vulnerable individuals) made non-household contacts, suggesting shielding and self-isolating individuals needed greater support to further reduce their number of interactions and to minimise infection risk.

Participants who were self-isolating as a precautionary measure, or after having been contacted by Test and Trace, reported fewer contacts than those not shielding or self-isolating. However, participants self-isolating due to experiencing symptoms or when a member of their household had symptoms did not have reduced contact rate, possibly due to the small number of participants reporting these circumstances. Participants who reported 'not sure' as their COVID circumstance had a significantly lower non-household contact rate than those not self-isolating or shielding. This may have been due to a pause in shielding guidance coinciding with the release of the survey, which may have left participants unsure of their current circumstance.[36–38]

This survey captured the point in time where cases were starting to consistently rise for the first time since March 2020, with the reproduction number estimated to be between 0.8 and 1.1.[15,39–41] The level of social mixing in the UK at the time of this survey enabled epidemic growth.

This study was likely subject to recruitment bias, as the survey was online and open to anyone living in the UK with no active recruitment process. The survey was under-represented by children, teenagers, young adults and the very elderly, as well as ethnic minorities. In particular, underrepresentation of the very elderly (80+) limited our ability to gain insight into mixing patterns of the age group at highest risk of severe COVID-19 disease. Additionally, as we asked participants to report their contact rate, the study may have suffered from recall bias. If a participant reported meeting 15 or more contacts, information was asked about their contacts collectively rather than as individual interactions. When grouping contacts into age groups, participants could select up to '20+' contacts for each age group, which may have led to us underestimating some participant's non-household contact rates; see supplementary materials. Participants were asked about their current COVID circumstance and contact behaviour for consecutive days (contacts were those made the previous day), which may bias the association of contact rate with COVID circumstance. Comparisons to pre-pandemic contact levels in the UK are based on social contact studies conducted within the UK prior to 2020, however, these are subject to differences in study population and study design in particular sample distributions and data collection methods.


For peer review only - http://bmjopen.bmj.com/site/about/guidelines.xhtml

**Acknowledgements**

We would like to thank the participants of the study for providing their time and information, and Prof Julia Gog OBE and Rev Richard Coles for helping to promote the survey.

**Contributions**

JREB, CPJ and JMR all conceived and designed the study. JREB conducted the analysis and wrote the first draft of the manuscript. All authors edited the manuscript.

**Data Availability Statement**

Data are available in a public, open access repository.

**Ethics Statements**

**Patient consent for publication**

Not applicable.

**Ethics approval**

This study was approved by the Faculty of Health and Medicine Ethics Committee at Lancaster University (reference FHMREC19135). Participation in the study was voluntary, with each participant (and where appropriate parent or guardian) giving their consent before proceeding.

**Competing Interests**

We declare we have no competing interests.

**Funding**

JREB is supported by a Lancaster University Faculty of Health and Medicine doctoral scholarship. JMR and CPJ were supported by UKRI through the JUNIPER modelling consortium [grant number MR/V038613/1].

For peer review only

## References


1 Cabinet Office. Staying at home and away from others (social distancing). Cabinet Office. 2020.https://www.gov.uk/government/publications/full-guidance-on-staying-at-home-and-away-from-others/full-guidance-on-staying-at-home-and-away-from-others (accessed Aug 2020).

2 Department of Health and Social Care. Making a support bubble with another household. 2020.https://www.gov.uk/guidance/making-a-support-bubble-with-another-household (accessed Sep 2020).

3 Department for Transport. Coronavirus (COVID-19): travel corridors. 2020.https://www.gov.uk/guidance/coronavirus-covid-19-travel-corridors (accessed Sep 2020).

4 GOV UK. Register your establishment for the Eat Out to Help Out Scheme. Gov.uk. 2020.https://www.gov.uk/guidance/register-your-establishment-for-the-eat-out-to-help-out-scheme (accessed Feb 2021).

5 Cabinet Office. Staying alert and safe (social distancing). 2020.https://www.gov.uk/government/publications/staying-alert-and-safe-social-distancing (accessed Aug 2020).

6 NI direct government services. Coronavirus (COVID-19). 2020.https://www.nidirect.gov.uk/campaigns/coronavirus-covid-19 (accessed Sep 2020).

7 Scottish government. Coronavirus (COVID-19) Phase 3: Scotland's route map update. 2020.https://www.gov.scot/publications/coronavirus-covid-19-framework-decision-making-scotlands-route-map-through-out-crisis-phase-3-update/pages/8/ (accessed Sep 2020).

8 Welsh Government. Coronavirus (COVID-19) social distancing guidance for everyone in Wales. 2020.https://gov.wales/coronavirus-social-distancing-guidance (accessed Sep 2020).

9 Wallinga J, Teunis P, Kretzschmar M. Using data on social contacts to estimate age-specific transmission parameters for respiratory-spread infectious agents. *Am J Epidemiol* 2006;**164**:936–44. doi:10.1093/aje/kwj317

10 Read JM, Edmunds WJ, Riley S, *et al.* Close encounters of the infectious kind: methods to measure social mixing behaviour. *Epidemiol Infect* 2012;**140**:2117–30. doi:10.1017/S0950268812000842

11 Mossong J, Hens N, Jit M, *et al.* Social contacts and mixing patterns relevant to the spread of infectious diseases. *PLoS Med* 2008;**5**:e74. doi:10.1371/journal.pmed.0050074

12 Danon L, Read JM, House TA, *et al.* Social encounter networks: characterizing Great Britain. *Proc Biol Sci* 2013;**280**:20131037. doi:10.1098/rspb.2013.1037

13 Klepac P, Kucharski AJ, Conlan AJK, *et al.* Contacts in context: large-scale setting-specific social mixing matrices from the BBC Pandemic project. Epidemiology. 2020.

14 Jarvis CI, Van Zandvoort K, Gimma A, *et al.* Quantifying the impact of physical distance measures on the transmission of COVID-19 in the UK. *BMC Med* 2020;**18**:124. doi:10.1186/s12916-020-01597-8


For peer review only

15 Public Health England. Coronavirus (COVID-19) in the UK. 2020.https://coronavirus.data.gov.uk (accessed Aug 2020).

[dataset] 16 Bridgen J, Jewell C, Read J. CoCoNet manuscript data. 2021.http://www.research.lancs.ac.uk/portal/en/datasets/coconet-manuscript-data(52d69555-0092-4757-808b-997939cdcfc0).html

17 Office for National Statistics. Estimates of the population for the UK, England and Wales, Scotland and Northern Ireland, 2020. 2020.https://www.ons.gov.uk/peoplepopulationandcommunity/populationandmigration/populationestimates/datasets/populationestimatesforukenglandandwalesscotlandandnorthernireland (accessed Aug 2020).

18 Office for National Statistics. Families and households. 2019.https://www.ons.gov.uk/peoplepopulationandcommunity/birthsdeathsandmarriages/families/datasets/familiesandhouseholdsfamiliesandhouseholds (accessed Aug 2020).

19 The R Project for Statistical Computing. https://www.r-project.org/ (accessed 19 Aug 2020).

20 Nishiura H, Oshitani H, Kobayashi T, *et al.* Closed environments facilitate secondary transmission of coronavirus disease 2019 (COVID-19). *MedRxiv* Published Online First: 2020.https://www.medrxiv.org/content/10.1101/2020.02.28.20029272v1.abstract

21 Eames KTD, Tilston NL, Brooks-Pollock E, *et al.* Measured dynamic social contact patterns explain the spread of H1N1v influenza. *PLoS Comput Biol* 2012;**8**:e1002425. doi:10.1371/journal.pcbi.1002425

22 Jarvis CI, Gimma A, van Zandvoort K, *et al.* The impact of local and national restrictions in response to COVID-19 on social contacts in England: a longitudinal natural experiment. *BMC Med* 2021;**19**:52. doi:10.1186/s12916-021-01924-7

23 Liu CY, Berlin J, Kiti MC, *et al.* Rapid review of social contact patterns during the COVID-19 pandemic. *Epidemiology* 2021;**32**. doi:10.1097/EDE.0000000000001412

24 Coletti P, Wambua J, Gimma A, *et al.* CoMix: comparing mixing patterns in the Belgian population during and after lockdown. *Sci Rep* 2020;**10**:21885. doi:10.1038/s41598-020-78540-7

25 Zhang J, Litvinova M, Liang Y, *et al.* Changes in contact patterns shape the dynamics of the COVID-19 outbreak in China. *Science* 2020;**368**:1481–6. doi:10.1126/science.abb8001

26 Latsuzbaia A, Herold M, Bertemes J-P, *et al.* Evolving social contact patterns during the COVID-19 crisis in Luxembourg. *PLoS One* 2020;**15**:e0237128. doi:10.1371/journal.pone.0237128

27 GOV UK. Prime Minister's statement on coronavirus (COVID-19): 17 July 2020. 2020.https://www.gov.uk/government/speeches/pm-statement-on-coronavirus-17-july-2020 (accessed 2021).

28 Sze S, Pan D, Nevill CR, *et al.* Ethnicity and clinical outcomes in COVID-19: A systematic review and meta-analysis. *EClinicalMedicine* 2020;**29**:100630. doi:10.1016/j.eclinm.2020.100630

29 Public Health England. Disparities in the risk and outcomes of COVID-19. 2020.

For peer review only

30 Office for National Statistics. Updating ethnic contrasts in deaths involving the coronavirus (COVID-19), England and Wales: deaths occurring 2 March to 28 July 2020. ONS. 2020.https://www.ons.gov.uk/peoplepopulationandcommunity/birthsdeathsandmarriages/deaths/articles/updatingethniccontrastsindeathsinvolvingthecoronaviruscovid19englandandwales/latest (accessed Jan 2021).

31 Fancourt D, Bu F, Mak HW, *et al.* Covid-19 Social Study Results Release 25. *UCL* 2020.

32 Office for National Statistics. Which occupations have the highest potential exposure to the coronavirus (COVID-19)? 2020.https://www.ons.gov.uk/employmentandlabourmarket/peopleinwork/employmentandemployeetypes/articles/whichoccupationshavethehighestpotentialexposuretothecoronaviruscovid19/2020-05-11 (accessed Aug 2020).

33 European Centre for Disease Prevention and Control. COVID-19 clusters and outbreaks in occupational settings in the EU/EEA and the UK. 2020.https://www.ecdc.europa.eu/en/publications-data/covid-19-clusters-and-outbreaks-occupational-settings-eueea-and-uk (accessed 2021).

34 Industrial Injuries Advisory Council. COVID-19 and occupation: position paper 48. 2021.

35 Smith LE, Potts HWW, Amlot R, *et al.* Adherence to the test, trace and isolate system: results from a time series of 21 nationally representative surveys in the UK (the COVID-19 Rapid Survey of Adherence to Interventions and Responses [CORSAIR] study). *MedRxiv* Published Online First: 2020. doi:10.1101/2020.09.15.20191957

36 Department of Health and Social Care. Updates to the shielding programme on national and regional levels. 2020.https://www.gov.uk/government/news/updates-to-the-shielding-programme-on-national-and-regional-levels (accessed Sep 2020).

37 NI direct government services. Coronavirus (COVID-19): pausing of shielding for extremely vulnerable people. 2020.https://www.nidirect.gov.uk/articles/coronavirus-covid-19-pausing-shielding-extremely-vulnerable-people (accessed Sep 2020).

38 Scottish government. Shielding to be paused. 2020.https://www.gov.scot/news/shielding-to-be-paused/ (accessed Sep 2020).

39 Children's Task and Finish Group. Update on Children, Schools and Transmission. 2020.https://www.gov.uk/government/publications/tfc-children-and-transmission-4-november-2020 (accessed May 2021).

40 Scientific Advisory Group for Emergencies. SPI-M-O: Consensus statement on COVID-19, 12 August 2020. Gov.uk. 2020.https://www.gov.uk/government/publications/spi-m-o-consensus-statement-on-covid-19-12-august-2020 (accessed Jun 2021).

41 Scientific Advisory Group for Emergencies. SPI-M-O Consensus Statement on COVID-19, 19 August 2020. Gov.uk. 2020.https://assets.publishing.service.gov.uk/government/uploads/system/uploads/attachment_data/file/916883/spi-m-o-consensus-statement-s0702-sage-52-200819.pdf (accessed Jun 2021).

For peer review only

**Figure Legends**

**Figure 1.** (A) Age and sex distribution of participants with ONS 2019 mid-year estimates. (B) Degree distribution of non-zero contacts. (C) Distribution of reported non-zero contact rate by age group. Note, log scale of x-axis in B and C.

**Figure 2.** Adjusted incidence rate ratios for number of non-household contacts reported for selected variables.

For peer review only


For peer review only - http://bmjopen.bmj.com/site/about/guidelines.xhtml

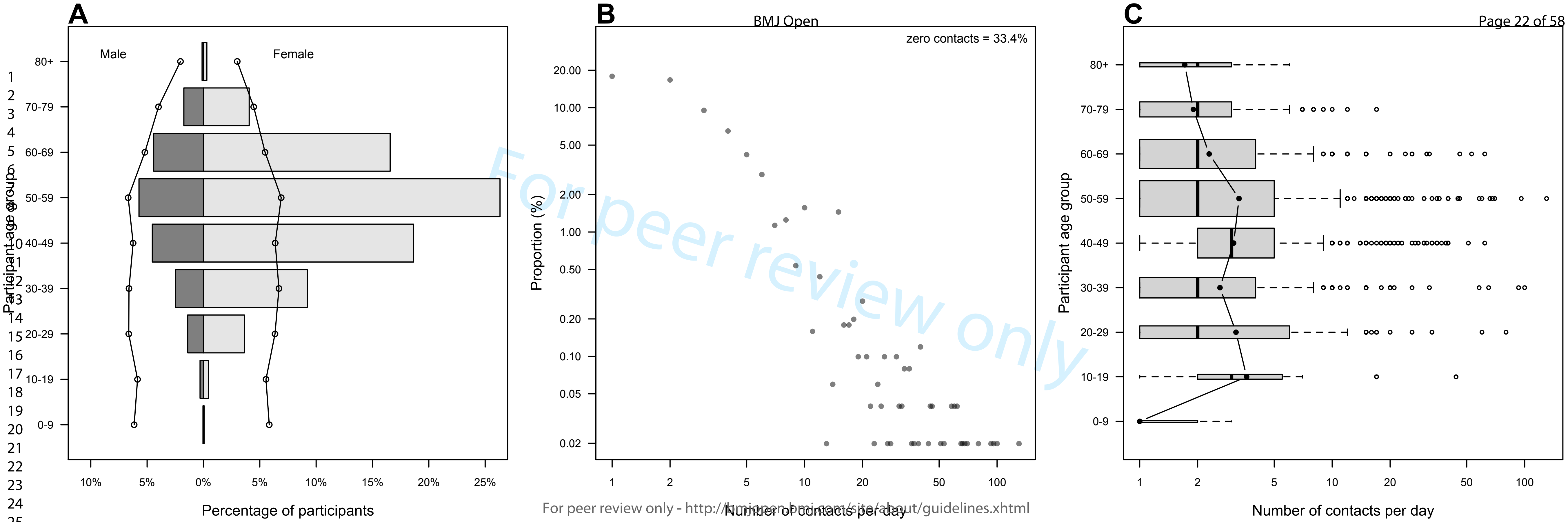


For peer review only

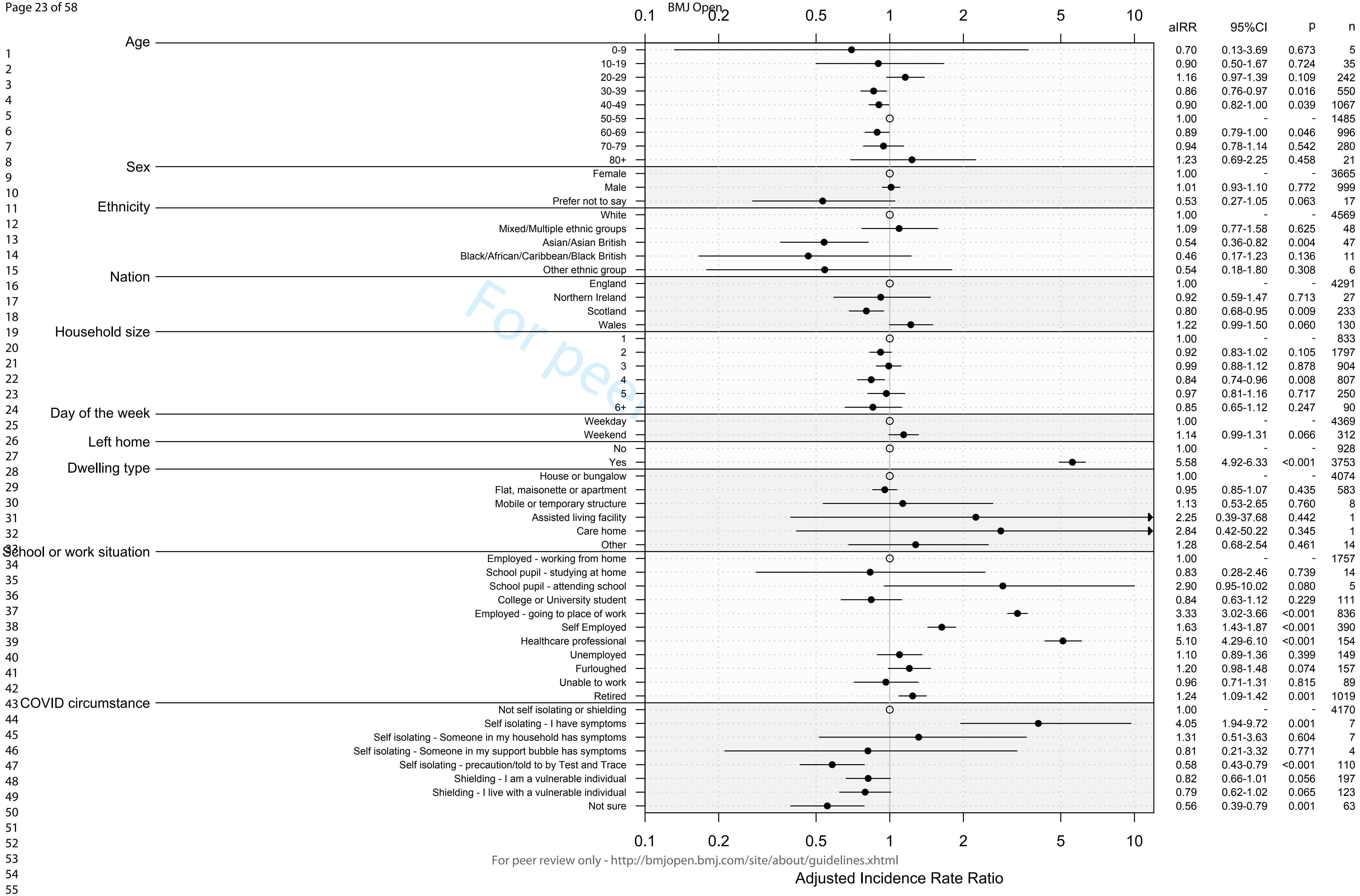


For peer review only - http://bmjopen.bmj.com/site/about/guidelines.xhtml

# Supplementary Material: Social mixing patterns in the UK following the relaxation of COVID-19 pandemic restrictions: a cross-sectional online survey

Jessica RE Bridgen; Lancaster Medical School, Lancaster University, Lancaster, UK, LA1 4YW
Chris P Jewell; Lancaster Medical School, Lancaster University, Lancaster, UK, LA1 4YW
Jonathan M Read; Lancaster Medical School, Lancaster University, UK, LA1 4YW
jonathan.read@lancs.ac.uk (corresponding author)

## Contents

*Analysis* ....................................................................................................................2
Age-specific mixing rates ....................................................................................................2
Variables associated with variation in non-household contact rate - model selection ....................2
Contact clustering or transitivity ..........................................................................................2
Visiting other households ....................................................................................................3
Household visits ...................................................................................................................3
Support bubbles ...................................................................................................................3
Survey methodology - limitations .........................................................................................3
*Figures* ....................................................................................................................*4*
*Tables* ....................................................................................................................*5*
*References* ....................................................................................................................*19*
*Survey Questions* ....................................................................................................................*20*

For peer review only

## Analysis

### Age-specific mixing rates

To calculate age-specific mixing patterns we first defined matrix $C_{ij}$, where $C_{ij}$ was the number of non-household contacts reported between participant age groups *i* and contact age groups *j*. The mean contact rate per age group ($M_{ij}$) was given by

$$M_{ij} = \frac{C_{ij}}{N_i}$$

where $N_i$ was the number of participants in age group *i*. As a measure of uncertainty, we calculated confidence intervals by taking 1,000 bootstrapped samples of participants.

Similarly, age-specific non-household contact rates were derived from the POLYMOD data.[1] We calculated the percentage decrease of age-specific non-household contact rates between the POLYMOD data and the CoCoNet data; Figure 1.

We found moderate assortative mixing by age, in line with both current and pre-pandemic contact studies (q = 0.38, 95%CI 0.17 to 0.58); Figure 1. Of all ages under 80 years old, 30-39 year olds had the highest non-household contact rate with those aged 80 or over; 4.8% (95%CIs 3.77 to 6.04) of non-household contacts reported by 30-39 years olds were with someone aged 80+.

### Variables associated with variation in non-household contact rate - model selection

To identify characteristics of the participant associated with their rate of daily non-household contact we fitted a negative binomial model to the daily number of non-household contacts reported by participants. We then used our selected explanatory variables as candidate variables for a forward stepwise model selection process.

Participant age group, sex, ethnicity, working situation and COVID circumstance were included as explanatory variables in all models. Candidate explanatory variables were: nation of residence (England, Northern Ireland, Scotland or Wales); household size; dwelling type; whether the contact reporting day was a weekend or week day; whether the participant had left their home on the contact reporting day.

The model selection process selected the following variables from candidate variables: age, sex, ethnicity, working situation, COVID circumstance, nation, household size, day of the week, whether the participant had left their home as explanatory variables. Dwelling type was not selected as an explanatory variable. Their association with the rate of non-household contact in the fully adjusted model is shown in Table 3.

### Contact clustering or transitivity

Participants who made fewer than 15 contacts and did not live alone were asked if anyone in their household had met each of their contacts that same day, as a measure of clustering (also called transitivity) within social networks.[2] We estimated 40.4% (95%CI 39.1 to 41.6) of non-household contacts were also met by another household member on the same day. The proportion of contacts encountered by participant and household members was highest if the contact was under 20 years old: contact aged 0 to 4 (77.5%, 95%CI 66.8 to 86.1); aged 5



For peer review only - http://bmjopen.bmj.com/site/about/guidelines.xhtml

to 9 (80.0%, 95%CI 72.7 to 86.1); aged 10 to 19 (63.2%, 95%CI 56.1 to 69.9). This indicates that non-household interactions with children tend to be made with multiple individuals from those households.

**Visiting other households**

Evidence suggests that transmission of COVID-19 often occurs within households.[3] We found that 12.2% (95%CI 11.3 to 13.1) of participants reported visiting another household. Females (aOR 1.2, 95%CI 0.98 to 1.56) and members of support bubbles (aOR 1.92, 95%CI 1.61 to 2.28) were more likely to have visited another household; Table 7.

**Household visits**

The mean rate of contacts made with non-household members (non-household contacts) by those not leaving their home was 0.4 $d^{-1}$ (95%CI 0.4 to 0.5), with 23.8% (95%CI 21.2 to 26.6) meeting one or more non-household contacts. This contact rate was significantly lower than for those who did leave their home (incidence rate ratio (IRR) 0.12, 95%CI 0.11 to 0.14, p-value <0.001).

**Support bubbles**

A substantial proportion (40.1%, 95%CI 38.7 to 41.4) of participants reported being part of a support bubble, with 43.1% (95%CI 40.9 to 45.3) joining with a single-person household. Males were less likely to be part of a support bubble (aOR 0.68, 95%CI 0.58 to 0.78); support bubble membership was not associated with age group or ethnicity. Support bubbles had a median (non-participant side) size of 2.0 (25th percentile 1.0, 75th percentile 3.0) and mean size of 2.2, (95%CI 2.1 to 2.2), and were mostly encountered two or fewer days in the past week.

**Survey methodology - limitations**

When calculating the mean number of daily non-household contacts, an assumption for the maximum number of contacts was made. The survey asked participants how many contacts they had made yesterday, with the option of '0 to 15 or more'. If participants selected '15 or more', they were asked to group the contacts they had made by age, by selecting an integer between 0 and 19 or '20+' for each contact age category. To calculate the mean number of contacts, where '20+' contacts was selected, this was assumed to be 20 contacts. We may, therefore, have underestimated the maximum number of contacts of some participants and non-household contact rates.



For peer review only - http://bmjopen.bmj.com/site/about/guidelines.xhtml

**Figures**

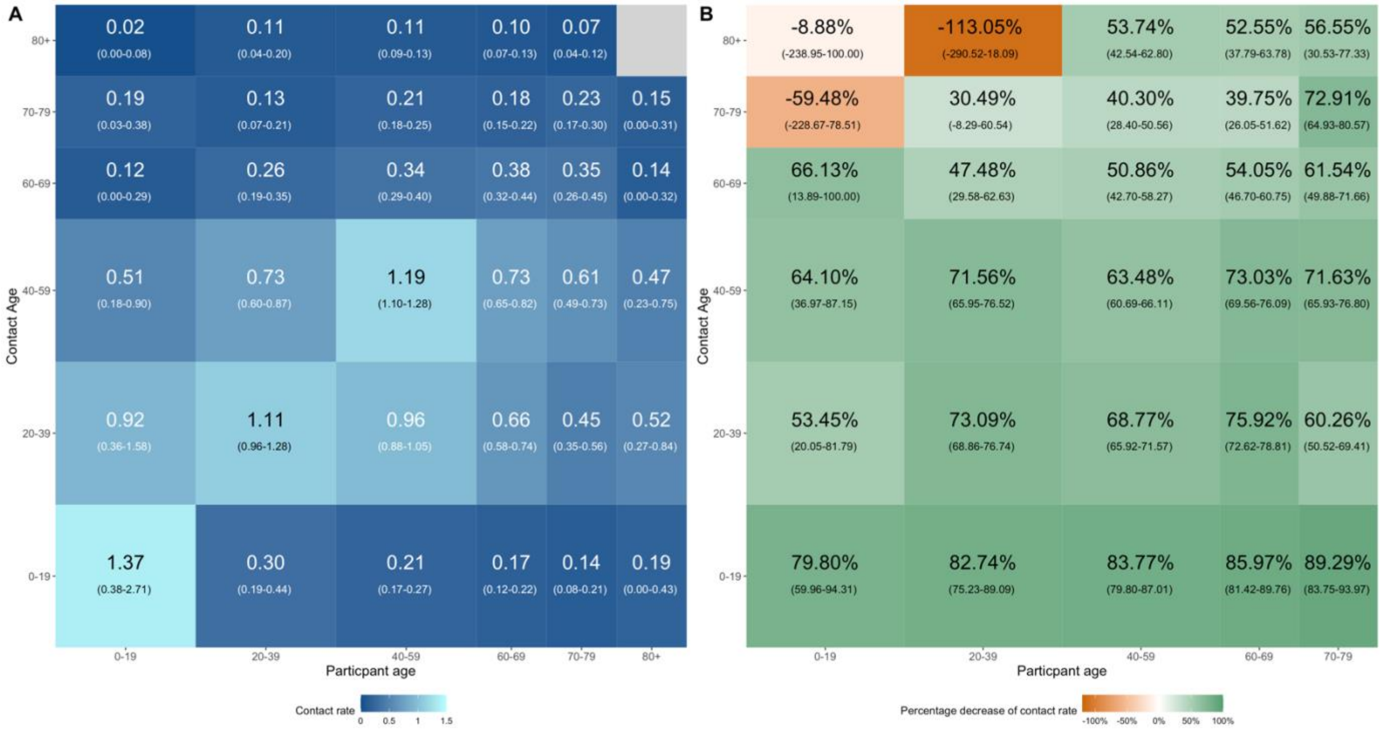


**Figure 1.** (A) Mean non-household contact rate (number of contacts per day) with different age groups reported by participant age group; bootstrapped 95% confidence intervals shown in parentheses. (B) Percentage decrease of non-household contact rate between the POLYMOD data (2005-2006) and the CoCoNet data; bootstrapped 95% confidence intervals shown in parentheses.



For peer review only - http://bmjopen.bmj.com/site/about/guidelines.xhtml

## Tables

**Table 1.** Comparison of non-household contact rates across different UK contact surveys.

| | Mean contact rate, $d^{-1}$ (25th and 75th percentile) | | | | |
|---|---|---|---|---|---|
| Study, contact type and geography of sample | **CoCoNet Number of participants** | **CoCoNet non-household contacts UK** | **CoMix non-household contacts UK** [4] | **Social Contact Survey non-household contacts, Great Britain** [5] | **POLYMOD, non-household contacts, Great Britain** [1] |
| Sampling period | | 28 July 2020 to 14 August 2020 Non-lockdown period | 24 March 2020 to 27 March 2020 Lockdown period | 2009 Pre-pandemic | 2005-2006 Pre-pandemic |
| All participants | 5,037 | 2.9 (0, 3) | 1.4 (0, 1) | 25.9 (5, 23) | 9.6 (4, 13) |
| Age group | | | | | |
| 0-9 | 5 | 1.0 (0, 1) | - | 29.8 (7, 46) | 8.9 (3, 13) |
| 10-19 | 37 | 3.6 (0, 3) | - | 43.1 (8, 40) | 12.3 (5, 18) |
| 20-29 | 250 | 3.2 (0, 3) | 1.1 (0, 1)* | 29.0 (7, 27) | 9.7 (5, 13) |
| 30-39 | 594 | 2.6 (0, 3) | 1.4 (0, 1) | 25.4 (6, 25) | 8.9 (4, 12) |
| 40-49 | 1167 | 3.1 (0, 4) | 1.4 (0, 1) | 30.8 (6, 29) | 9.8 (4, 13) |
| 50-59 | 1617 | 3.3 (0, 3) | 1.6 (0, 2) | 28.6 (6, 26) | 8.2 (3, 11) |
| 60-69 | 1056 | 2.3 (0, 3) | 1.4 (0, 2) | 23.0 (4, 18) | 8.2 (4, 11) |
| 70-79 | 290 | 1.9 (0, 3) | 1.1 (1, 1)** | 19.1 (3, 17) | 6.8 (3, 11) |
| 80+ | 21 | 1.7 (0, 2) | - | 13.2 (1,10) | - |
| Sex† | | | | | |
| Female | 3978 | 2.8 (0, 3) | 1.4 (0, 1) | 27.5 (5, 26) | 10.2 (4, 14) |
| Male | 1037 | 2.9 (0, 3) | 1.3 (0, 1) | 22.6 (4, 19) | 9.0 (4, 13) |
| Prefer not to say | 22 | 1.5 (0, 2) | - | - | - |
| Household size | | | | | |
| 1 | 875 | 2.8 (0, 3) | 1.6 (1, 2) | 24.3 (4, 20) | 7.5 (3, 11) |
| 2 | 1902 | 2.6 (0, 3) | 1.5 (0, 2) | 23.7 (5, 21) | 9.2 (4, 12) |
| 3 | 979 | 3.2 (0, 3) | 1.2 (0, 1) | 24.6 (5, 25) | 9.6 (4, 14) |
| 4 | 896 | 2.7 (0, 3) | 1.4 (0, 1) | 33.4 (6, 33) | 10.0 (4, 14) |
| 5 | 284 | 3.2 (0, 4) | 1.1 (0, 1) | 30.7 (7, 30) | 10.9 (5, 15) |
| 6+ | 101 | 3.8 (0, 4) | 1.1 (0, 1) | 45.6 (8, 36) | 10.1 (5, 15) |

* CoMix age group 18-29
** CoMix age group 70+
† Comix and POLYMOD report participants' gender rather than sex


For peer review only - http://bmjopen.bmj.com/site/about/guidelines.xhtml

**Table 2.** Adjusted incidence rate ratios for number of daily non-household contacts by select variables. Intercept of 0.41 (95 %CI 0.35-0.48). Dispersion parameter of 1.07 (95%CI 1.00-1.14)

| | **Multivariable analysis[1]** | |
|---|---|---|
| | **aIRR (95%CI)** | **p-value** |
| Age | | |
| 0-9 | 0.70 (0.13-3.69) | 0.673 |
| 10-19 | 0.90 (0.50-1.67) | 0.724 |
| 20-29 | 1.16 (0.97-1.39) | 0.109 |
| 30-39 | 0.86 (0.76-0.97) | 0.016 |
| 40-49 | 0.90 (0.82-1.00) | 0.039 |
| 50-59 | 1.00 | - |
| 60-69 | 0.89 (0.79-1.00) | 0.046 |
| 70-79 | 0.94 (0.78-1.14) | 0.542 |
| 80+ | 1.23 (0.69-2.25) | 0.458 |
| Sex | | |
| Female | 1.00 | - |
| Male | 1.01 (0.93-1.10) | 0.772 |
| Prefer not to say | 0.53 (0.27-1.05) | 0.063 |
| Ethnicity | | |
| White | 1.00) | - |
| Mixed/Multiple ethnic groups | 1.09 (0.77-1.58) | 0.625 |
| Asian/Asian British | 0.54 (0.36-0.82) | 0.004 |
| Black/African/Caribbean/Black British | 0.46 (0.17-1.23) | 0.136 |
| Other ethnic group | 0.54 (0.18-1.80) | 0.308 |
| Nation | | |
| England | 1.00 | - |
| Northern Ireland | 0.92 (0.59-1.47) | 0.713 |
| Scotland | 0.80 (0.68-0.95) | 0.009 |
| Wales | 1.22 (0.99-1.50) | 0.060 |
| Household size | | |
| 1 | 1.00 | - |
| 2 | 0.92 (0.83-1.02) | 0.105 |



For peer review only - http://bmjopen.bmj.com/site/about/guidelines.xhtml

| | Multivariable analysis[1] | |
|---|---|---|
| | aIRR (95%CI) | p-value |
| 3 | 0.99 (0.88-1.12) | 0.878 |
| 4 | 0.84 (0.74-0.96) | 0.008 |
| 5 | 0.97 (0.81-1.16) | 0.717 |
| 6+ | 0.85 (0.65-1.12) | 0.247 |
| Day of the week (contacts recorded) | | |
| Weekday | 1.00 | - |
| Weekend | 1.14 (0.99-1.31) | 0.066 |
| Left home yesterday | | |
| No | 1.00 | - |
| Yes | 5.58 (4.92-6.33) | <0.001 |
| Dwelling type | | |
| House or bungalow | 1.00 | - |
| Flat, maisonette or apartment | 0.95 (0.85-1.07) | 0.435 |
| Mobile or temporary structure | 1.13 (0.53-2.65) | 0.760 |
| Assisted living facility | 2.25 (0.39-37.68) | 0.442 |
| Care home | 2.84 (0.42-50.22) | 0.345 |
| Other | 1.28 (0.68-2.54) | 0.461 |
| School or work situation | | |
| Employed - working from home | 1.00 | - |
| School pupil - studying at home | 0.83 (0.28-2.46) | 0.739 |
| School pupil - attending school | 2.90 (0.95-10.02) | 0.080 |
| College or University student | 0.84 (0.63-1.12) | 0.229 |
| Employed - going to place of work | 3.33 (3.02-3.66) | <0.001 |
| Self Employed | 1.63 (1.43-1.87) | <0.001 |
| Healthcare professional | 5.10 (4.29-6.10) | <0.001 |
| Unemployed | 1.10 (0.89-1.36) | 0.399 |
| Furloughed | 1.20 (0.98-1.48) | 0.074 |
| Unable to work | 0.96 (0.71-1.31) | 0.815 |
| Retired | 1.24 (1.09-1.42) | 0.001 |
| COVID-19 circumstance | | |
| Not self isolating or shielding | 1.00 | - |

For peer review only

| | Multivariable analysis[1] | |
|---|---|---|
| | aIRR (95%CI) | p-value |
| Self isolating - I have symptoms | 4.05 (1.94-9.72) | 0.001 |
| Self isolating - Someone in my household has symptoms | 1.31 (0.51-3.63) | 0.604 |
| Self isolating - Someone in my support bubble has symptoms | 0.81 (0.21-3.32) | 0.771 |
| Self isolating - precaution/told to by Test and Trace | 0.58 (0.43-0.79) | <0.001 |
| Shielding - I am a vulnerable individual | 0.82 (0.66-1.01) | 0.056 |
| Shielding - I live with a vulnerable individual | 0.79 (0.62-1.02) | 0.065 |
| Not sure | 0.56 (0.39-0.79) | 0.001 |

[1]Adjusted for age,sex, ethnicity, nation, household size, day of the week, left home, dwelling type, school or work situation and COVID-19 circumstance.
*This increased contact rate is due to one participant who was self-isolating with symptoms reporting a large number of contacts (*see results*).

For peer review only

**Table 3.** Adjusted incidence rate ratios for number of daily non-household contacts by select variables. Model intercept was 0.40 (95 %CI 0.34-0.47). Dispersion parameter of 1.07 (95%CI 1.00-1.13)

| | **Multivariable analysis[1]** | |
|---|---|---|
| | **aIRR (95%CI)** | **p-value** |
| Age | | |
| 0-9 | 0.69 (0.13-3.65) | 0.663 |
| 10-19 | 0.88 (0.49-1.64) | 0.688 |
| 20-29 | 1.14 (0.96-1.36) | 0.142 |
| 30-39 | 0.86 (0.76-0.97) | 0.012 |
| 40-49 | 0.90 (0.82-0.99) | 0.032 |
| 50-59 | 1.00 | - |
| 60-69 | 0.89 (0.79-1.00) | 0.047 |
| 70-79 | 0.95 (0.79-1.15) | 0.598 |
| 80+ | 1.24 (0.69-2.27) | 0.446 |
| Sex | | |
| Female | 1.00 | - |
| Male | 1.01 (0.93-1.11) | 0.762 |
| Prefer not to say | 0.53 (0.27-1.05) | 0.061 |
| Ethnicity | | |
| White | 1.00 | - |
| Mixed/Multiple ethnic groups | 1.08 (0.76-1.56) | 0.667 |
| Asian/Asian British | 0.53 (0.35-0.81) | 0.003 |
| Black/African/Carribean/Black British | 0.46 (0.17-1.23) | 0.135 |
| Other ethnic group | 0.53 (0.17-1.77) | 0.290 |
| Nation | | |
| England | 1.00 | - |
| Northern Ireland | 0.92 (0.59-1.47) | 0.708 |
| Scotland | 0.79 (0.67-0.94) | 0.006 |
| Wales | 1.23 (1.00-1.51) | 0.052 |
| Household size | | |
| 1 | 1.00 | - |
| 2 | 0.92 (0.83-1.02) | 0.133 |



For peer review only - http://bmjopen.bmj.com/site/about/guidelines.xhtml

| | Multivariable analysis[1] | |
|---|---|---|
| | aIRR (95%CI) | p-value |
| 3 | 1.00 (0.89-1.13) | 0.981 |
| 4 | 0.85 (0.75-0.96) | 0.010 |
| 5 | 0.98 (0.83-1.17) | 0.839 |
| 6+ | 0.88 (0.68-1.16) | 0.357 |
| Day of the week (contacts recorded) | | |
| Weekday | 1.00 | - |
| Weekend | 1.14 (0.99-1.31) | 0.074 |
| Left home yesterday | | |
| No | 1.00 | |
| Yes | 5.60 (4.94-6.36) | <0.001 |
| School or work situation | | |
| Employed - working from home | 1.00 | - |
| School pupil - studying at home | 0.84 (0.29-2.48) | 0.752 |
| School pupil - attending school | 2.96 (0.97-10.23) | 0.074 |
| College or University student | 0.86 (0.65-1.14) | 0.287 |
| Employed - going to place of work | 3.32 (3.02-3.66) | <0.001 |
| Self Employed | 1.63 (1.43-1.87) | <0.001 |
| Healthcare professional | 5.05 (4.25-6.03) | <0.001 |
| Unemployed | 1.11 (0.90-1.37) | 0.354 |
| Furloughed | 1.20 (0.98-1.48) | 0.074 |
| Unable to work | 0.96 (0.71-1.30) | 0.798 |
| Retired | 1.24 (1.09-1.42) | 0.001 |
| COVID-19 circumstance | | |
| Not self-isolating or shielding | 1.00 | - |
| Self-isolating - I have symptoms* | 4.07 (1.96-9.79) | 0.001 |
| Self-isolating - Someone in my household has symptoms | 1.30 (0.51-3.60) | 0.614 |
| Self-isolating - Someone in my support bubble has symptoms | 0.82 (0.21-3.34) | 0.780 |
| Self-isolating - precaution/told to do so by Test and Trace | 0.58 (0.43-0.79) | <0.001 |
| Shielding - I am a vulnerable individual | 0.81 (0.66-1.01) | 0.050 |


For peer review only - http://bmjopen.bmj.com/site/about/guidelines.xhtml

| | Multivariable analysis[1] | |
|---|---|---|
| | aIRR (95%CI) | p-value |
| Shielding - I live with a vulnerable individual | 0.79 (0.62-1.01) | 0.063 |
| Not sure | 0.55 (0.39-0.78) | 0.001 |

[1]Adjusted for age, sex, ethnicity, nation, household size, day of the week, left home, school or work situation and COVID-19 circumstance.
*This increased contact rate is due to one participant who was self-isolating with symptoms reporting a high number of contacts (*see results*).

For peer review only

**Table 4.** Characteristics of participants who reported 'Self isolating' or 'Shielding' as their COVID circumstance. N is the number of participants who provided a response to the question.

| | Number of self-isolating participants (%) | Number of shielding participants (%) | Number of participants not self-isolating or shielding (%) |
|---|---|---|---|
| Age group | N = 136 | N = 353 | N = 4,511 |
| 0-9 | 0 (0.0%) | 0 (0.0%) | 5 (0.1%) |
| 10-19 | 1 (0.7%) | 4 (1.1%) | 33 (0.7%) |
| 20-29 | 7 (5.1%) | 12 (3.4%) | 233 (5.2%) |
| 30-39 | 11 (8.1%) | 28 (7.9%) | 549 (12.2%) |
| 40-49 | 17 (12.5%) | 64 (18.1%) | 1093 24.2%) |
| 50-59 | 41 (30.1%) | 95 (26.9%) | 1465 (32.5%) |
| 60-69 | 32 (23.5%) | 104 (29.5%) | 905 (20.1%) |
| 70-79 | 22 (16.2%) | 43 (12.2%) | 215 (4.8%) |
| 80+ | 5 (3.7%) | 3 (0.8%) | 13 (0.3%) |
| No response | 0 (0.0%) | 0 (0.0%) | 0 (0.0%) |
| Sex | N = 136 | N = 353 | N = 4,511 |
| Female | 111 (81.6%) | 286 (81.0%) | 3548 (78.7%) |
| Male | 25 (18.4%) | 65 (18.4%) | 943 (20.9%) |
| Prefer not to say | 0 (0.0%) | 2 (0.6%) | 20 (0.4%) |
| No response | 0 (0.0%) | 0 (0.0%) | 0 (0.0%) |
| Ethnicity | N = 136 | N = 353 | N = 4,511 |
| White | 126 (92.6%) | 334 (94.6%) | 4336 (96.1%) |
| Mixed/Multiple ethnic groups | 5 (3.7%) | 8 (2.3%) | 36 (0.8%) |
| Asian/Asian British | 3 (2.2%) | 2 (0.6%) | 43 (1.0%) |
| Black/African/Caribbean/Black British | 0 (0.0%) | 2 (0.6%) | 9 (0.2%) |
| Other ethnic group | 1 (0.7%) | 0 (0.0%) | 6 (0.1%) |
| Prefer not to say | 0 (0.0%) | 3 (0.8%) | 13 (0.3%) |
| No response | 1 (0.7%) | 4 (1.1%) | 68 (1.5%) |
| Left home yesterday | N = 135 | N = 350 | N = 4,495 |
| No | 64 (47.4%) | 145 (41.4%) | 778 (17.3%) |
| Yes | 71 (52.6%) | 205 (58.6%) | 3717 (82.7%) |

For peer review only

| | Number of self-isolating participants (%) | Number of shielding participants (%) | Number of participants not self-isolating or shielding (%) |
|---|---|---|---|
| No response | 0 (0.0%) | 0 (0.0%) | 0 (0.0%) |
| Part of a support bubble | N = 136 | N= 352 | N = 4,505 |
| No | 87 (64.0%) | 238 (67.6%) | 2664 (59.1%) |
| Yes | 49 (36.0%) | 114 (32.4%) | 1841 (40.9%) |
| No response | 0 (0.0%) | 0 (0.0%) | 0 (0.0%) |
| Social distancing | N = 53 | N= 167 | N = 2,989 |
| Yes, all of the time | 39 (73.6%) | 117 (70.1%) | 1728 (57.8%) |
| More than half of the time | 10 (18.9%) | 36 (21.6%) | 877 (29.3%) |
| Less than half of the time | 4 (7.5%) | 11 (6.6%) | 278 (9.3%) |
| No, none of the time | 0 (0.0%) | 3 (1.8%) | 86 (2.9%) |
| Not sure | 0 (0.0%) | 0 (0.0%) | 20 (0.7%) |
| No response | 0 (0.0%) | 0 (0.0%) | 0 (0.0%) |
| | **Mean daily non-household contact rate (IQR)** | | |
| | **Sef-isolating participants** | **Shielding participants** | **Participants not shielding or self-isolating** |
| | N = 134 | N = 348 | N = 4,484 |
| All participants | 1.2 (0, 2) | 1.3 (0, 2) | 3.1 (0, 3) |

review only

**Table 5.** Adjusted incidence rate ratios for number of daily non-household contacts by select variables. Self-isolating individual with large number of contacts removed for this analysis (see results). Intercept of 0.41 (95 %CI 0.35-0.48). Dispersion parameter of 1.07 (95%CI 1.00-1.14)

| | **Multivariable analysis[1]** | |
|---|---|---|
| | **aIRR (95%CI)** | **p-value** |
| Age | | |
| 0-9 | 0.69 (0.13-3.64) | 0.662 |
| 10-19 | 0.89 (0.49-1.64) | 0.691 |
| 20-29 | 1.15 (0.97-1.39) | 0.114 |
| 30-39 | 0.86 (0.76-0.98) | 0.021 |
| 40-49 | 0.91 (0.82-1.00) | 0.044 |
| 50-59 | 1.00 | - |
| 60-69 | 0.89 (0.79-1.00) | 0.047 |
| 70-79 | 0.94 (0.78-1.14) | 0.540 |
| 80+ | 1.23 (0.69-2.25) | 0.457 |
| Sex | | |
| Female | 1.00 | - |
| Male | 1.02 (0.93-1.11) | 0.730 |
| Prefer not to say | 0.53 (0.27-1.05) | 0.063 |
| Ethnicity | | |
| White | 1.00) | - |
| Mixed/Multiple ethnic groups | 1.13 (0.79-1.62) | 0.511 |
| Asian/Asian British | 0.54 (0.36-0.82) | 0.004 |
| Black/African/Caribbean/Black British | 0.47 (0.17-1.23) | 0.137 |
| Other ethnic group | 0.54 (0.18-1.79) | 0.305 |
| Nation | | |
| England | 1.00 | - |
| Northern Ireland | 0.92 (0.59-1.47) | 0.713 |
| Scotland | 0.80 (0.68-0.95) | 0.009 |
| Wales | 1.22 (1.00-1.50) | 0.059 |
| Household size | | |
| 1 | 1.00 | - |

For peer review only

| | Multivariable analysis[1] | |
|---|---|---|
| | aIRR (95%CI) | p-value |
| 2 | 0.92 (0.83-1.02) | 0.105 |
| 3 | 0.98 (0.87-1.11) | 0.805 |
| 4 | 0.84 (0.74-0.95) | 0.007 |
| 5 | 0.97 (0.81-1.15) | 0.692 |
| 6+ | 0.85 (0.65-1.12) | 0.244 |
| Day of the week (contacts recorded) | | |
| Weekday | 1.00 | - |
| Weekend | 1.14 (0.99-1.31) | 0.068 |
| Left home yesterday | | |
| No | 1.00 | - |
| Yes | 5.54 (4.89-6.29) | <0.001 |
| Dwelling type | | |
| House or bungalow | 1.00 | - |
| Flat, maisonette or apartment | 0.95 (0.85-1.07) | 0.414 |
| Mobile or temporary structure | 1.21 (0.56-2.87) | 0.647 |
| Assisted living facility | 2.25 (0.39-37.46) | 0.441 |
| Care home | 2.78 (0.41-48.81) | 0.355 |
| Other | 1.28 (0.68-2.54) | 0.460 |
| School or work situation | | |
| Employed - working from home | 1.00 | - |
| School pupil - studying at home | 0.85 (0.29-2.50) | 0.763 |
| School pupil - attending school | 2.90 (0.95-10.00) | 0.080 |
| College or University student | 0.86 (0.65-1.15) | 0.291 |
| Employed - going to place of work | 3.34 (3.04-3.68) | <0.001 |
| Self Employed | 1.64 (1.43-1.87) | <0.001 |
| Healthcare professional | 5.12 (4.31-6.12) | <0.001 |
| Unemployed | 1.10 (0.89-1.37) | 0.369 |
| Furloughed | 1.21 (0.99-1.49) | 0.063 |
| Unable to work | 1.00 (0.74-1.35) | 0.993 |
| Retired | 1.25 (1.09-1.42) | 0.001 |
| COVID-19 circumstance | | |



For peer review only - http://bmjopen.bmj.com/site/about/guidelines.xhtml

| | Multivariable analysis[1] | |
|---|---|---|
| | aIRR (95%CI) | p-value |
| Not self isolating or shielding | 1.00 | - |
| Self isolating - I have symptoms | 0.83 (0.24-2.82) | 0.775 |
| Self isolating - Someone in my household has symptoms | 1.31 (0.51-3.62) | 0.606 |
| Self isolating - Someone in my support bubble has symptoms | 0.81 (0.21-3.30) | 0.768 |
| Self isolating - precaution/told to by Test and Trace | 0.58 (0.43-0.79) | <0.001 |
| Shielding - I am a vulnerable individual | 0.81 (0.66-1.00) | 0.050 |
| Shielding - I live with a vulnerable individual | 0.79 (0.62-1.02) | 0.065 |
| Not sure | 0.56 (0.39-0.79) | 0.001 |

[1] Adjusted for age,sex, ethnicity, nation, household size, day of the week, left home, dwelling type, school or work situation and COVID-19 circumstance.

For peer review only

**Table 6.** Association of participant characteristics and maintaining social distancing more than half of the time with contacts (adjusted odds ratios). N = 3058. Model intercept of 9.34 (7.24-12.20).

| | Adjusted Odds Ratio (95%CI)[1] | p-value |
|---|---|---|
| Age | | |
| 10-19 | 0.30 (0.08-1.25) | 0.077 |
| 20-29 | 0.62 (0.38-1.06) | 0.069 |
| 30-39 | 0.66 (0.46-0.95) | 0.024 |
| 40-49 | 0.88 (0.65-1.19) | 0.415 |
| 50-59 | 1.00 | - |
| 60-69 | 0.88 (0.61-1.28) | 0.496 |
| 70-79 | 0.91 (0.49 -1.79) | 0.778 |
| 80+ | 0.28 (0.08-1.30) | 0.064 |
| School or work situation | | |
| Employed - working from home | 1.00 | - |
| College or University student | 0.68 (0.33-1.55) | 0.335 |
| Employed - going to place of work | 0.71 (0.53-0.96) | 0.025 |
| Self Employed | 1.48 (0.92-2.51) | 0.126 |
| Healthcare professional | 0.26 (0.17-0.40) | <0.001 |
| Unemployed | 0.59 (0.35-1.07) | 0.068 |
| Furloughed | 1.01 (0.54-2.05) | 0.979 |
| Unable to work | 1.58 (0.56-6.61) | 0.453 |
| Retired | 1.40 (0.90-2.19) | 0.136 |

[1] Adjusted for age and school or work situation
* School pupils excluded from analysis due to insufficient data.

For peer review only

**Table 7.** Association of participant characteristics with risk (adjusted odds ratios) of visiting another household (N = 4,030). Model intercept 0.11 (0.09-0.14).

| | **Adjusted Odds Ratio (95%CI)**[1] | **p-value** |
|---|---|---|
| Age | | |
| 10-19 | 0.75 (0.18-2.20) | 0.643 |
| 20-29 | 0.77 (0.47-1.21) | 0.277 |
| 30-39 | 0.85 (0.62-1.15) | 0.313 |
| 40-49 | 1.03 (0.82-1.30 | 0.805 |
| 50-59 | 1.00 | - |
| 60-69 | 1.09 (0.86-1.38) | 0.479 |
| 70-79 | 1.18 (0.79-1.72) | 0.413 |
| 80+ | 0.73 (0.11-2.73) | 0.682 |
| Sex | | |
| Female | 1.23 (0.98-1.56) | 0.073 |
| Male | 1.00 | - |
| Prefer not to say | 1.07 (0.17-3.92) | 0.926 |
| Part of a support bubble | | |
| No | 1.00 | - |
| Yes | 1.92 (1.61-2.28) | <0.001 |

[1]Adjusted for age, sex and whether a participants was a part of a support bubble.
*0-9 year olds excluded from analysis due to insufficient data.

For peer review only

**References**

1 Mossong J, Hens N, Jit M, *et al.* Social contacts and mixing patterns relevant to the spread of infectious diseases. *PLoS Med* 2008;**5**:e74.

2 Wasserman S, Faust K, Stanley (University of Illinois Wasserman, Urbana-Champaign). *Social Network Analysis: Methods and Applications*. Cambridge University Press 1994.

3 Leclerc QJ, Fuller NM, Knight LE, *et al.* What settings have been linked to SARS-CoV-2 transmission clusters? *Wellcome Open Res* 2020;**5**:83.

4 Jarvis CI, Van Zandvoort K, Gimma A, *et al.* Quantifying the impact of physical distance measures on the transmission of COVID-19 in the UK. *BMC Med* 2020;**18**:124.

5 Danon L, Read JM, House TA, *et al.* Social encounter networks: characterizing Great Britain. *Proc Biol Sci* 2013;**280**:20131037.

For peer review only

**Survey Questions - Round 1**

Q1
Are you aged 13 or over?

◯ Yes

◯ No

Q1.a
**Please make sure you agree to the following before continuing with the survey:**
You currently live in the UK;
You have read the Participant Information Sheet and fully understand what is expected of you within this study;
Your participation is voluntary and you are aware that you can stop the survey at any point; You understand that data submitted prior to closing the survey will be collected;
You consent to Lancaster University keeping the anonymised data for a period of 10-years after the study has finished;
If you are filling out the survey on behalf of someone else, please make sure you have their consent before continuing.

◯ **I consent to taking part in the CoCoNet study**

Q1.b
**If you are under the age of 13 we do ask that a parent or guardian fills out the survey with you.**

Please take the time to read through and discuss the *Information sheet for Children* together. The parent or guardian should also read through the more detailed *Participant Information Sheet*.

**Please make sure you both agree to the following before continuing with the survey:**
I live in the UK;
I have read and understood the information sheet(s);
I understand I can stop the survey at any point;
I understand that my answers will be kept for 10 years after the study has finished.

| | Child | Parent/Guardian |
|---|---|---|
| **I agree to take part in the CoCoNet study/ I consent to my child taking part in the study** | ☐ | ☐ |

For peer review only

Q2 Where in the UK do you currently live?

- ◯ England
- ◯ Northern Ireland
- ◯ Scotland
- ◯ Wales
- ◯ I do not live in the UK

For peer review only

Q3 What is your age?

- ◯ 0 - 9 years old
- ◯ 10 - 19 years old
- ◯ 20 - 29 years old
- ◯ 30 - 39 years old
- ◯ 40 - 49 years old
- ◯ 50 - 59 years old
- ◯ 60 - 69 years old
- ◯ 70 - 79 years old
- ◯ Aged 80 or over

Q4 What is your sex?
*The answer you give can be different from what is on your birth certificate.*

- ◯ Female
- ◯ Male
- ◯ Prefer not to say

Q5 Which of the following best describes your ethnicity?

- ◯ English / Welsh / Scottish / Northern Irish / British
- ◯ Irish
- ◯ Gypsy or Irish Traveller
- ◯ Any other White background
- ◯ White and Black Caribbean
- ◯ White and Black African
- ◯ White and Asian
- ◯ Any other Mixed / Multiple ethnic background
- ◯ Indian
- ◯ Pakistani
- ◯ Bangladeshi
- ◯ Chinese
- ◯ Any other Asian background
- ◯ African
- ◯ Caribbean
- ◯ Any other Black / African / Caribbean background
- ◯ Arab
- ◯ Any other ethnic group
- ◯ Prefer not to say

For peer review only

Q6 What is the first part of your home postcode?
*For example, if your home postcode was LA1 4YW then you would enter LA1.*

_______________________________________________

Q7 Which type of accommodation best describes your home?

- ○ Flat, maisonette or apartment
- ○ House or bungalow
- ○ Mobile or temporary structure
- ○ Assisted living facility
- ○ Care home
- ○ Other

For peer review only

Q8
What is your current school or work situation?

- ◯ School pupil - studying at home
- ◯ School pupil - still attending school
- ◯ College or University student
- ◯ Employed - working from home
- ◯ Employed - still going to place of work
- ◯ Self Employed
- ◯ Healthcare professional
- ◯ Unemployed
- ◯ Furloughed
- ◯ Unable to work
- ◯ Retired
- ◯ Other

Q9 Currently, do you regularly meet members of the general public as part of your job?

- ◯ Yes
- ◯ No

For peer review only

Q10
Are you self-isolating or shielding because of COVID-19?
*A vulnerable individual here refers to a clinically extremely vulnerable person.*

- ◯ I am not self-isolating or shielding
- ◯ Self Isolating - I have symptoms of COVID
- ◯ Self Isolating - Someone in my household has symptoms of COVID
- ◯ Self Isolating - Someone in my support bubble has symptoms of COVID
- ◯ Self Isolating - As a precaution / told to do so by Test and Trace
- ◯ Shielding - I am a vulnerable individual
- ◯ Shielding - I live with a vulnerable individual
- ◯ Not sure

Q11
How many other people currently live with you at home?

- ◯ 0 - I live alone
- ◯ 1
- ◯ 2
- ◯ 3
- ◯ 4
- ◯ 5 or more

For peer review only

Q12
How many people of each age group live with you at home?
*Do not include yourself.*

*Drop down options of 0, 1, 2, 3, 4, 5 or more for each age group.*

0 - 9 year olds
10 - 19 year olds
20 - 29 year olds
30 - 39 year olds
40 - 49 year olds
50 - 59 year olds
60 -69 year olds
70 - 79 year olds
Aged 80 or over

Q13
Have you formed a support bubble with another household?
*A single-person household can join with one other household and interact without maintaining social distance.*

- ○ Yes
- ○ No

Q14
How many people of each age group are part of your support bubble?
*Do not include your own household members.*

*Drop down options of 0, 1, 2, 3, 4, 5 or more for each age group.*

0 - 9 year olds
10 - 19 year olds
20 - 29 year olds
30 - 39 year olds
40 - 49 year olds
50 - 59 year olds
60 -69 year olds
70 - 79 year olds
Aged 80 or over

For peer review only

Q15
Thinking about the past 7 days, on how many of these days did you meet someone from your support bubble?

- ◯ None
- ◯ 1 day
- ◯ 2 days
- ◯ 3 days
- ◯ 4 days
- ◯ 5 days
- ◯ 6 days
- ◯ 7 days
- ◯ Not sure

For peer review only

Q16

Did you leave your home or property yesterday?

*Do not include going into your private garden, but do include visits to shared or communal gardens or spaces.*

- ◯ Yes
- ◯ No

Q17

Where did you go yesterday? Tick all that apply.

- ☐ Visited the home of someone else
- ☐ My school or workplace
- ☐ Doctor's surgery or healthcare facility
- ☐ Supermarket or convenience store
- ☐ Other shops or retail spaces (e.g. garden centre, clothing shops, drive-through food outlets)
- ☐ Restaurant, café or pub
- ☐ For a walk or exercise
- ☐ Other - *please do not include any identifying information*

_____________________________________________

For peer review only

Q18
What modes of transport did you use yesterday? Tick all that apply.

- ☐ I walked or cycled
- ☐ I travelled in a car by myself
- ☐ I travelled in a car with another person(s)
- ☐ I took a bus, tram or train
- ☐ I took an aeroplane or ferry

For peer review only


For peer review only - http://bmjopen.bmj.com/site/about/guidelines.xhtml

Q19
Not including those that you live with, how many people did you meet yesterday?
*Only include those you had a face-to-face conversation with.*

- ○ None
- ○ 1
- ○ 2
- ○ 3
- ○ 4
- ○ 5
- ○ 6
- ○ 7
- ○ 8
- ○ 9
- ○ 10
- ○ 11
- ○ 12
- ○ 13
- ○ 14
- ○ 15 or more

For peer review only

Q20
Please tell us about each of the people you met yesterday.
*Information collected for up to 14 contacts.*

| How old were they? *Please estimate the person's age if you are unsure.* | | | | | | | | Did you meet this person indoors? *For example, in a shop, office or house.* | | Did anyone that you live with also meet this person yesterday? | | |
|---|---|---|---|---|---|---|---|---|---|---|---|---|
| 0 - 4 | 5 - 9 | 10 - 19 | 20 - 39 | 40 - 59 | 60 - 69 | 70 - 79 | 80 + | Yes | No | Yes | No | Not sure |

Q21
Please tell us about each of the people you met yesterday.
*Information collected for up to 14 contacts.*

| How old were they? *Please estimate the person's age if you are unsure.* | | | | | | | | Did you meet this person indoors? *For example, in a shop, office or house.* | |
|---|---|---|---|---|---|---|---|---|---|
| 0 - 4 | 5 - 9 | 10 - 19 | 20 - 39 | 40 - 59 | 60 - 69 | 70 - 79 | 80 + | Yes | No |

For peer review only

Q22
How many people of each age group did you meet yesterday?
*Drop down choice of integers 1 - 19 or '20 or more' for each age group.*

0-4 year olds

5-9 year olds

10-19 year olds

20-39 year olds

40-59 year olds

60-69 year olds

70-79 year olds

Aged 80 or over

Q23
Did you meet these people indoors or outdoors?
*For example, meeting someone indoors could be in a shop, office or house etc.*

- I met everyone indoors
- I met most people indoors
- I met most people outdoors
- I met everyone outdoors
- Not sure

For peer review only

Q24
Were you able to maintain social distance from everyone you met yesterday?
*Do not include people that you live with or those in your support bubble. Please refer to the government advice for the recommended social distance in your area.*

- ◯ Yes, all of the time
- ◯ More than half of the time
- ◯ Less than half of the time
- ◯ No, none of the time
- ◯ Not sure

Q25 Of the people you live with, how many people stayed at home all day yesterday?

- ◯ None
- ◯ 1
- ◯ 2
- ◯ 3
- ◯ 4
- ◯ 5 or more
- ◯ Not sure

For peer review only

Q26 Thinking about the past 7 days, on how many of these days did you leave your home or property?

- ◯ None
- ◯ 1 day
- ◯ 2 days
- ◯ 3 days
- ◯ 4 days
- ◯ 5 days
- ◯ 6 days
- ◯ 7 days
- ◯ Not sure

Q27 What was the furthest distance from home you travelled over the past 7 days?

- ◯ Less than 2 miles (3 km)
- ◯ 2 - 9 miles (3 - 15 km)
- ◯ 10 - 19 miles (16 - 31 km)
- ◯ 20 - 49 miles (32 - 79 km)
- ◯ 50 miles (80 km) or more

For peer review only

STROBE Statement—Checklist of items that should be included in reports of ***cross-sectional studies***

| | Item No | Recommendation | Page No |
|---|---|---|---|
| **Title and abstract** | 1 | (*a*) Indicate the study's design with a commonly used term in the title or the abstract | 1 |
| | | (*b*) Provide in the abstract an informative and balanced summary of what was done and what was found | 2 |
| **Introduction** | | | |
| Background/rationale | 2 | Explain the scientific background and rationale for the investigation being reported | 4 |
| Objectives | 3 | State specific objectives, including any prespecified hypotheses | 5 |
| **Methods** | | | |
| Study design | 4 | Present key elements of study design early in the paper | 4/5 |
| Setting | 5 | Describe the setting, locations, and relevant dates, including periods of recruitment, exposure, follow-up, and data collection | 4/5 |
| Participants | 6 | (*a*) Give the eligibility criteria, and the sources and methods of selection of participants | 4/5 |
| Variables | 7 | Clearly define all outcomes, exposures, predictors, potential confounders, and effect modifiers. Give diagnostic criteria, if applicable | 5 |
| Data sources/ measurement | 8* | For each variable of interest, give sources of data and details of methods of assessment (measurement). Describe comparability of assessment methods if there is more than one group | 5/6 |
| Bias | 9 | Describe any efforts to address potential sources of bias | 5 |
| Study size | 10 | Explain how the study size was arrived at | 4 |
| Quantitative variables | 11 | Explain how quantitative variables were handled in the analyses. If applicable, describe which groupings were chosen and why | 5/6 |
| Statistical methods | 12 | (*a*) Describe all statistical methods, including those used to control for confounding | 5/6 |
| | | (*b*) Describe any methods used to examine subgroups and interactions | 6 |
| | | (*c*) Explain how missing data were addressed | 6 |
| | | (*d*) If applicable, describe analytical methods taking account of sampling strategy | NA |
| | | (*e*) Describe any sensitivity analyses | NA |
| **Results** | | | |
| Participants | 13* | (a) Report numbers of individuals at each stage of study—eg numbers potentially eligible, examined for eligibility, confirmed eligible, included in the study, completing follow-up, and analysed | 6/7/8 /10/11 |
| | | (b) Give reasons for non-participation at each stage | 7/8/10/11 |
| | | (c) Consider use of a flow diagram | NA |
| Descriptive data | 14* | (a) Give characteristics of study participants (eg demographic, clinical, social) and information on exposures and potential confounders | 6 |
| | | (b) Indicate number of participants with missing data for each variable of interest | 7/8/10/11 |
| Outcome data | 15* | Report numbers of outcome events or summary measures | 7/8/9/10/11 |



For peer review only

For peer review only - http://bmjopen.bmj.com/site/about/guidelines.xhtml

| | | | |
|---|---|---|---|
| Main results | 16 | (*a*) Give unadjusted estimates and, if applicable, confounder-adjusted estimates and their precision (eg, 95% confidence interval). Make clear which confounders were adjusted for and why they were included | 15/16 |
| | | (*b*) Report category boundaries when continuous variables were categorized | NA |
| | | (*c*) If relevant, consider translating estimates of relative risk into absolute risk for a meaningful time period | NA |
| Other analyses | 17 | Report other analyses done—eg analyses of subgroups and interactions, and sensitivity analyses | 12/13 |
| **Discussion** | | | |
| Key results | 18 | Summarise key results with reference to study objectives | 14/15 |
| Limitations | 19 | Discuss limitations of the study, taking into account sources of potential bias or imprecision. Discuss both direction and magnitude of any potential bias | 15 |
| Interpretation | 20 | Give a cautious overall interpretation of results considering objectives, limitations, multiplicity of analyses, results from similar studies, and other relevant evidence | 15 |
| Generalisability | 21 | Discuss the generalisability (external validity) of the study results | 15 |
| **Other information** | | | |
| Funding | 22 | Give the source of funding and the role of the funders for the present study and, if applicable, for the original study on which the present article is based | 16 |

*Give information separately for exposed and unexposed groups.

**Note:** An Explanation and Elaboration article discusses each checklist item and gives methodological background and published examples of transparent reporting. The STROBE checklist is best used in conjunction with this article (freely available on the Web sites of PLoS Medicine at http://www.plosmedicine.org/, Annals of Internal Medicine at http://www.annals.org/, and Epidemiology at http://www.epidem.com/). Information on the STROBE Initiative is available at www.strobe-statement.org.

For peer review only


For peer review only - http://bmjopen.bmj.com/site/about/guidelines.xhtml
